# Drivers of the spatiotemporal patterns of the mangrove crab metacommunity in a tropical bay

**DOI:** 10.1002/ece3.10191

**Published:** 2023-06-14

**Authors:** Xuan Gu, Guogui Chen, Yufeng Lin, Wenqing Wang, Mao Wang

**Affiliations:** ^1^ Key Laboratory of the Ministry of Education for Coastal and Wetland Ecosystems, College of the Environment & Ecology Xiamen University Xiamen China; ^2^ Zhangjiang Estuary Mangrove Wetland Ecosystem Station, National Observation and Research Station for the Taiwan Strait Marine Ecosystem Xiamen University Zhangzhou China; ^3^ Engineering Research Center of Fujian Province for Coastal Wetland Protection and Ecological Recovery, College of the Environment & Ecology Xiamen University Xiamen China; ^4^ State Key Laboratory of Water Environmental Simulation, School of Environment Beijing Normal University Beijing China; ^5^ Research and Development Center for Watershed Environmental Eco‐Engineering, Advanced Institute of Natural Sciences Beijing Normal University Zhuhai China

**Keywords:** element of metacommunity structure, functional diversity, mangrove crabs, metacommunity patterns, seasonal variation, variation partitioning analysis

## Abstract

Revealing community patterns and driving forces is essential in community ecology and a prerequisite for effective management and conservation efforts. However, the mangrove ecosystem and its important fauna group such as the crabs, still lack multi‐processes research under metacommunity framework, resulting in evidence and theorical application gaps. To fill these gaps, we selected China's most representative mangrove bay reserve in tropical zone as a stable experimental system and conducted a seasonal investigation (July 2020, October 2020, January 2021, and April 2021) of mangrove crabs. We performed a multi‐approach analysis using both pattern‐based and mechanistic method to distinguish the processes driving the mangrove crab metacommunity. Our results showed that the crab metacommunity exhibits a Clementsian pattern in the bay‐wide mangrove ecosystem but is influenced by both local environmental heterogeneity and spatial processes, thus representing a combined paradigm of species sorting and mass effect. Moreover, the long‐distance spatial constraints are more pronounced compared to the local environmental factors. This is reflected in the greater importance of the broad‐scale Moran's Eigenvector Maps, the distance‐decay pattern of similarity, and the difference in beta diversity dominated by the turnover component. This pattern changes throughout the year, mainly due to changes in dominant functional groups caused by the stress of changes in water salinity and temperature induced by air temperature and precipitation. This research provides multi‐dimension research data and relevant analysis, offering clear evidence for understanding the patterns and related driving forces of crab metacommunity in tropical bay mangroves, and verifies the applicability of some general laws in the system. Future studies can address more diverse spatiotemporal scales, gaining a clearer understanding to serve the conservation of mangrove ecosystems and economically important fishery species.

## Graphical abstract

Evidence from the mangrove crab metacommunity in a tropical bay indicates that large benthic fauna, which rely on the passive larval dispersal, continue to be predominantly influenced by dispersal constraints and spatial processes. Moreover, local environmental factors remain crucial, primarily by shaping seasonal community structures and driving functional diversities under the impact of climatic conditions. This study enriches our current understanding and highlights the significance of employing multi‐process research approaches to better comprehend dynamics of crab taxa.

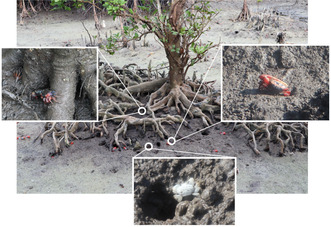

## 1 INTRODUCTION

The need for a comprehensive understanding of ecological problems in the face of global change has led to the exploration of multiple scales, with the metacommunity concept being a key framework in this endeavor (Colossi Brustolin et al., 2019; Lamy et al., 2021). A metacommunity refers to a collection of local communities that may interact through species dispersal and replacement (Leibold et al., 2004). The formation and stability of metacommunities are shaped by both the local selection process (such as niche differentiation and the environment filtering) and the spatial process (such as dispersal or colonization; Jabot et al., 2020; Leibold et al., 2004; Thompson et al., 2020). Hence, studying the patterns and mechanisms of metacommunities is crucial in gaining deeper insight into the driving forces of species diversity, distribution, and functional changes

With advancements in theory and technology, both mechanistic and pattern‐based frameworks have improved our understanding of metacommunities. Currently, the dominant mechanistic approach for studying metacommunities is based on decomposing the relative contributions of environmental filtering (effect of local environmental variables) and spatial factors (usually representing spatial structures generated by dispersal; Cottenie, 2005; Ovaskainen et al., 2019; Siqueira et al., 2012). This method helps distinguish different paradigms (including species sorting, mass effects, patch dynamics, and neutral dynamics, details in Appendix S1: Section S1) by evaluating the significance of environmental or spatial factors using a variation partitioning analysis (VPA) (Cottenie, 2005; Peres‐Neto et al., 2006; Peres‐Neto & Legendre, 2010). The pattern‐based approach, such as the elements of metacommunity structure (EMS) analysis, classifies metacommunities into multiple idealized patterns (details in Appendix S1: Section S2 and Figure S1) using three metrics: turnover, coherence, and boundary clumping, calculated from the site‐by‐species incidence matrices (Leibold & Mikkelson, 2002; Presley et al., 2010). However, over a period, community research often focuses solely on one approach of structure or mechanism, which can lead to vague inferences about ecological processes. The increasing number of research have recognized that patterns are often a combination of multiple ecological processes, including dispersal, random drift, environmental filtering, and biological interactions (Leibold et al., 2004, 2017; Logue et al., 2011). Consequently, studying metacommunities through combined structure and mechanism approaches has emerged as a new avenue and has been applied in numerous studies to date (e.g., Henry & Cumming, 2016; Li et al., 2021; Meynard et al., 2013). From a broader perspective, incorporating other metacommunity analysis methods such as distance‐decay relationship (Heino & Tolonen, 2017; Liu et al., 2022; Soininen et al., 2007), decomposition of beta diversity (Baselga, 2010; Soininen et al., 2018), and functional diversity analysis (Heino & Tolonen, 2017) into the research framework will offer a more multidimensional viewpoint

Nonetheless, empirical evidence from the field remains an indispensable foundation for community ecology theory. While the metacommunity concept strives to offer a comprehensive framework, the associated patterns and mechanisms exhibit spatiotemporal variability and are heavily contingent upon factors such as scale, system, and species characteristics. To date, extensive evidence has been amassed for a variety of organisms in different types of ecosystems. For example, plants (Meynard et al., 2013), birds (Henry & Cumming, 2016), and invertebrates (Siqueira et al., 2012) have been studied in terrestrial ecosystems (Marrec et al., 2018), while fishes (Heino et al., 2015), plankton (Diniz et al., 2021), and microorganisms (Wu et al., 2018) have been studied in freshwater (Heino et al., 2015) and marine ecosystems (Colossi Brustolin et al., 2019). Despite these advancements, there is a notable gap in research concerning the coastal intertidal zone, an ecotone uniquely characterized by the convergence of marine, freshwater, and terrestrial features. Consequently, the applicability of conclusions drawn from other ecosystems to the coastal intertidal zone is hindered by a lack of pertinent evidence, thus impeding our understanding. For instance, small organic metacommunities are predominantly influenced by deterministic processes (De Bie et al., 2012); lotic water systems are more affected by spatial processes (dispersal limitation), and lentic water bodies exhibit species sorting or Clementsian patterns (Heino et al., 2015); environmental heterogeneity and dispersal limitations jointly shape metacommunity patterns across various spatiotemporal scales (Cottenie, 2005).

Mangrove wetlands are highly interconnected and open coastal ecosystems, making them ideal study systems (Barbier et al., 2011; Duke et al., 2007; Wang et al., 2020). The constant flow of tidal water in and out of bays or estuaries promotes the rapid transport and exchange of nutrients and benthic propagules, connecting the local communities in the ecosystem and forming a typical metacommunity (Lee et al., 2014). Since mangrove wetlands are famous for their high degree of environmental heterogeneity (Carrasquilla‐Henao & Juanes, 2017; Leung, 2015; Wang et al., 2020), the combination of local selection and spatial process both has the potential to shape the species composition of the fauna metacommunity in mangrove ecosystems. However, despite its significance, the lack of relevant research leaves a gap in the evidence base

**FIGURE 1:**
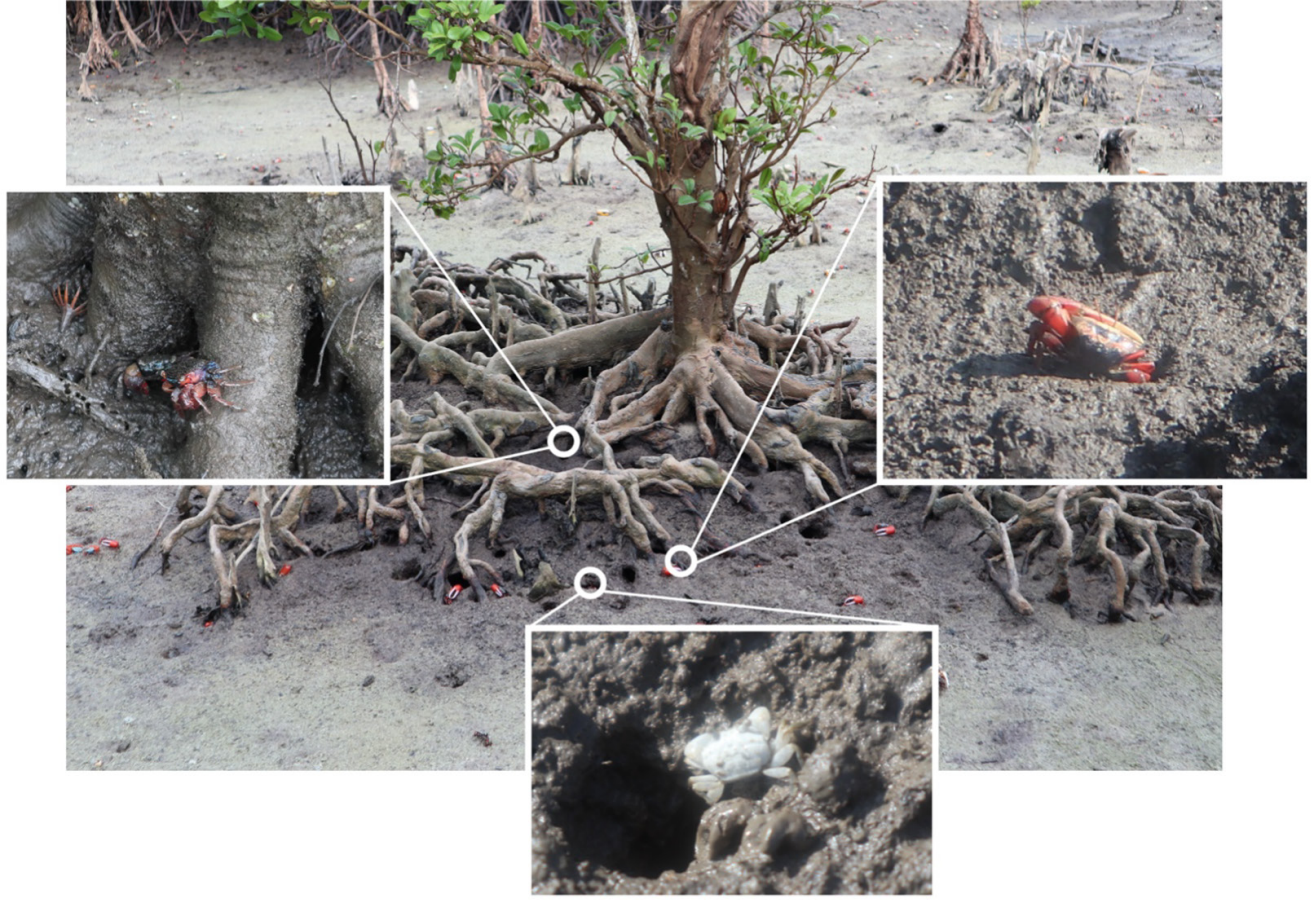
Crabs in mangrove ecosystem, left: *Parasesarma eumolpe*; right: *Tubuca arcuate*; bottom: *Ilyoplax serrata*. Photographer: Xuan Gu.

Crabs (Figure 1), which alter various ecological processes through burrowing and feeding, play a crucial role as ecosystem engineers in mangrove wetlands (Kristensen, 2008; Lee, 1998). Their terrestrial adaptation and the aquatic stage of their larvae grant them both the traits of local patch distribution and strong dispersal ability. The spatial and temporal distribution of the crab community influences the changes in community function and the impact on the mangrove ecosystem, making them a quintessential species among benthic faunas (Cannicci et al., 2008; Kristensen, 2008; Lee, 1998, 2008). At the local scale, numerous studies have been conducted to examine the effects of environmental heterogeneity (including salinity levels, vegetation density, temperature, sediment types, and oxygen levels) and biological interactions on various aspects of mangrove crab species (Cannicci et al., 2009, 2018; Diele et al., 2013; McLain & Pratt, 2010; Nobbs & Blamires, 2017; Wang et al., 2014; Xiang et al., 2020), such as diversity, biomass, larval development, behavior, and distribution. These findings underscore the significance of local selection in shaping mangrove crab metacommunities. However, research on multiscale processes encompassing bay, harbor, and regional scales remains limited, and further investigation is needed to determine the relative importance of dispersal and local environmental filtering

Here, we conducted this study to assess the relative importance of environmental and spatial processes in structuring a mangrove crab metacommunity. Our research aimed to address the following questions: (1) do spatial processes and local environment selection jointly influence the structure of the crab metacommunity, and what are the patterns of mangrove crab metacommunity structure at the bay‐wide scale, with particular attention to potential seasonal variations? (2) if taxonomic and functional diversity exhibit such changes, can they be ascribed to environmental factors linked to seasonal climatic fluctuations?

## 2 MATERIALS AND METHODS

### 2.1 Study system

**FIGURE 2:**
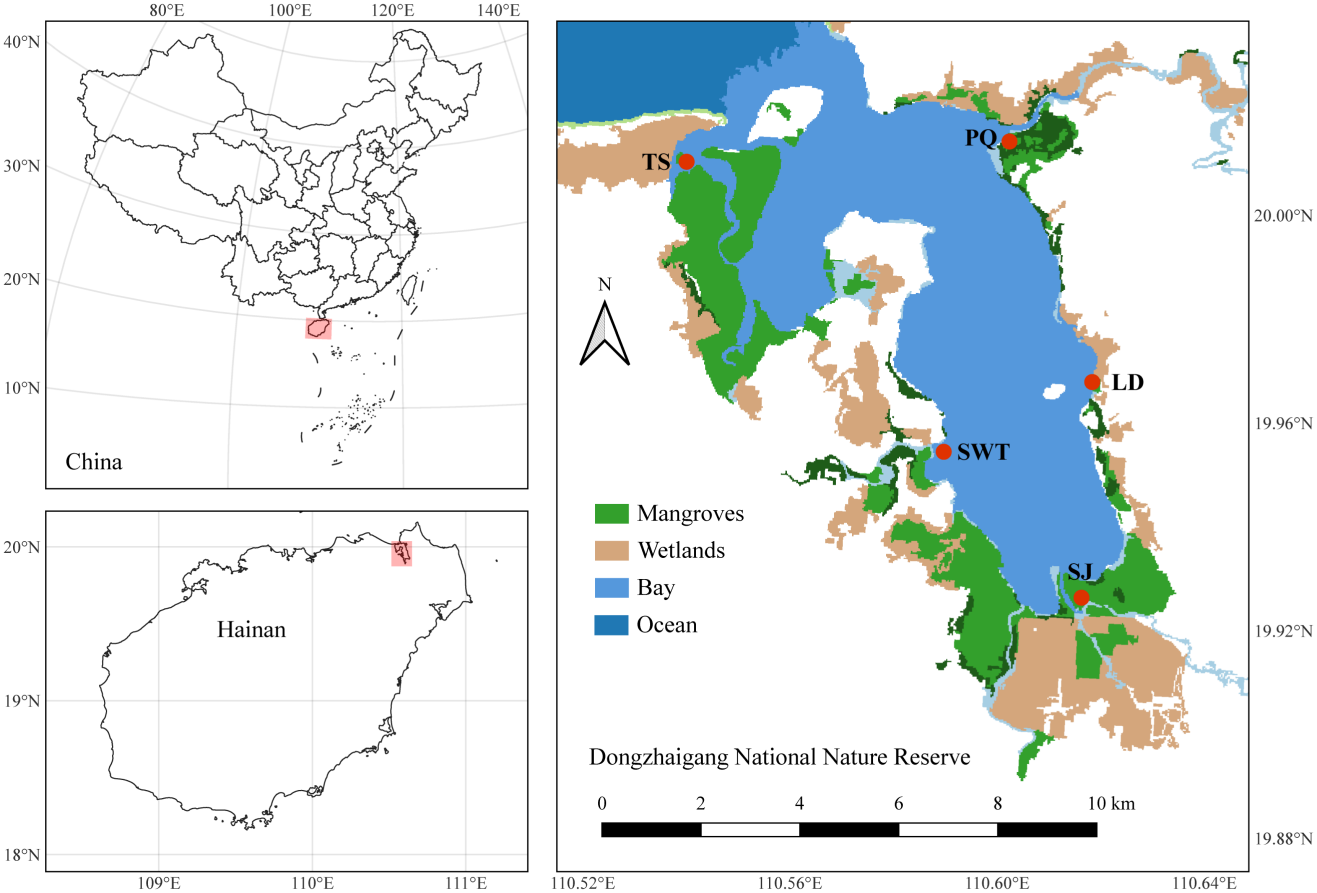
The distribution of sampling sites for the crab metacommunity within Dongzhaigang National Nature Reserve in Hainan Province, China. LD, Luodou; PQ, Puqian; SJ, Sanjiang; SWT, Shanweitou; TS, Tashi.

Our research was conducted in a tropical bay in Dongzhaigang National Nature Reserve (20°7′ N and 110°30′ E), which covers an area of 25 km^2^ (Figure 2). The semi‐enclosed and muddy‐bottom bay create a suitable habitat for mangrove forests and the fauna includes birds, fish, and benthos. The Dongzhaigang National Nature Reserve is characterized by several types of mangrove vegetation, each dominated separately by *Avicennia marina*, *Rhizophora stylosa*, *Bruguiera sexangular*, *Sonneratia apetala*, and *Ceriops tagal*. All these mangrove vegetation types, tidal flats, and tidal channels constitute the complex landscape connected by the flooding water. The irregular semidiurnal tide is the main tidal type, with mean tidal ranges from 1.6 to 1.8 m (Fu et al., 2019). During the sampling period, the monthly rainfall ranged from 11.6 to 419.9 mm with mean 111.5 mm, and the lowest temperature down to 6.1°C in winter and highest up to 38.4°C in summer with annual mean temperature as 24.3°C (Appendix S1: Figure S2). The climate data were extracted from the National Meteorological Science Data Center of China (http://data.cma.cn).

### 2.2 Crab metacommunity surveys in the mangroves area

The sampling was carried out over four seasons including summer (July 2020), autumn (October 2020), winter (January 2021), and spring (April 2021). We selected five sites, Tashi, Sanjiang, Shanweitou, Luodou, and Puqian, that represent the typical characteristics of different locations within the bay (Figure 2). These sites are spaced with an average of 7 km apart from each other, with the furthest distance being 12.6 km. To account for varied vegetation types and seasonal changes, 53 sampling quadrats (10 m × 10 m) were collected across the five study sites (9 at Luodou, 9 at Puqian, 11 at Sanjiang, 12 at Shanweitou, and 12 at Tashi) every season. The sampling quadrats were chosen to include various habitats such as tidal channels, tidal flats, and vegetation dominated by different mangrove plants. To conduct a quantitative survey, trap nets with a length of 10 m and 1 cm meshes were used. Three trap nets were placed simultaneously at each sampling quadrat as a repetition. The sampling was carried out for three consecutive days, once a day, to increase the sample size and account for any temporal variations in the crab population. The collection of crabs was carried out during low tide periods each day to maximize the chances of catching as many crabs as possible. The samples were then fixed in 4% formaldehyde and transferred to 75% ethanol after washing with water within 3 days. All crab specimens were identified at the species level following Shih et al. (2010) and the World Register of Marine Species (https://www.marinespecies.org). The weight of the collected crabs was measured using an electronic balance (BL‐1200F Setra™), while the carapace width was measured using an electronic vernier caliper (CD‐15AX, Mitutoyo™) in the lab.

The sampling was under the permission and supports provided by Hainan Dongzhaigang National Nature Reserve Authority. Ethical approval was not required for this study, and no licenses were necessary for the collection of crabs.

### 2.3 Aquatic environment factors and vegetation variables

To assess the environmental conditions influencing crab survival, activity, and larval development (Anger, 2003; Diele & Simith, 2006; Nurdiani & Zeng, 2007), five aquatic variables were measured in situ on the day when the highest spring tide level occurred during the crab sampling period for each season. These variables included pH, total dissolved solids (mg/L), dissolved oxygen (mg/L), salinity (‰), and water temperature (°C), and were recorded using a Multiline™ 3420 (WTW™) portable digital multiparameter. To minimize measurement error, three points were randomly selected within each sample quadrat (10 m × 10 m) for measuring each variable, and measurements were taken at each of the three points.

The vegetation variable was defined as the root type of the mangrove ecosystem, which reflects the complexity of the habitat and its influence on crab movement (Crowder & Cooper, 1982; Wang et al., 2014). The tidal‐flat and tidal channels were considered to have no roots, while *Avicennia marina* and *Sonneratia apetala* have pneumatophores, *Bruguiera sexangular* has knee roots, *Rhizophora stylosa* has prop roots, and *Ceriops tagal* has spreading roots. Further descriptions of each root type can be found in Appendix S1: Figure S3.

### 2.4 Moran eigenvectors maps as spatial factors

We constructed spatial predictors through the general form of Moran's Eigenvector Maps (MEM), which is based on Graph‐theory (Dray et al., 2006). It was calculated with spatial weighting matrix (W), which is the result of the Hadamard product of connectivity matrix (B) and edge weighting matrix (A). In this study, the B matrices were graph‐based with Delaunay triangulation criteria and calculated from Cartesian coordinates. To detect the non‐linear relationship instead of using equal weight in matrix A, we transformed the geographic distances to similarities with the concave‐down function (*f*_2_),

(1) $$s_{\mathrm{ij}}=1-{\left(d_{\mathrm{ij}}/\max\left(d_{\mathrm{ij}}\right)\right)}^{\alpha }$$

where *d*_*ij*_ is the geographic distance between plots *i* and *j*. *s*_*ij*_ is in the range [0, 1], and when *α* = 1, it is the linear function *f*_*1*_. Thus, we computed *f*_2_ from *α* = 1 to *α* = 10 and kept the best model with the lowest value of the Akaike information criterion (AIC). Before that, the abundance data of the crab community was detrended to minimize the spatially related environmental gradient and Hellinger‐transformed. The AIC was lowest when *α* = 2 (see Appendix S1: Figure S4), indicated a non‐linear spatial weight, and selected four MEMs (MEM2, MEM3, MEM5, MEM12) including both the broader scale and fine scale. The analysis was done with “spdep” (Bivand & Wong, 2018), “vegan” (Oksanen et al., 2020), and “adespatial” (Dray et al., 2021) packages in R software (version 4.1.0) (R Core Team, 2021).

### 2.5 Data analysis

All statistical analyses were conducted using R software (version 4.1.0; R Core Team, 2021).

#### 2.5.1 Sample adequacy

To assess if the mangrove crab sampling was representative and determine the optimal temporal scale to reflect the metacommunity structure, we used species accumulation curves (SACs) to test the completeness of the crab sampling. We built curves by using “specaccum” function from “vegan” (Oksanen et al., 2020) package in R with “random” method, which finds the mean SAC and its standard deviation from random permutations. The SACs of four seasons revealed that the effectiveness of sampling is different among seasons (Appendix S1: Figure S5). The SAC of the whole year is nearly converged meaning it is suited for analysis of the distribution pattern of crabs (Appendix S1: Figure S5). Based on these, we did the further analysis from Section 2.5.2 to Section 2.5.4 at annual level.

#### 2.5.2 Additive diversity partitioning

Gamma diversity can be additively partitioned into the sum of mean alpha and beta diversities of the same dimension (Lande, 1996). Additionally, Crist et al. (2003) then extended it across multiple scales in a hierarchical sampling design. For levels of sampling within *i* = 1, 2, … m, while the grain size is increasing from *i* = 1 to *i* = m, diversities within or among samples can be calculated as:

(2) $$\beta_{i=m}=\gamma -\alpha_{i=m};$$

(3) $$\beta_{i}=\alpha_{i+1}-\alpha_{i};$$

(4) $$\gamma =\alpha_{i=1}+\sum_{i=1}^{m}\beta_{i}.$$

Thus, the average *α*_*i*_ is calculated based on the weighted average value proportional to sample abundances (weight = “prop” in “adipart” function of “vegan” (Oksanen et al., 2020) package in R).

#### 2.5.3 Hierarchical and variation partitioning for redundancy analysis

To reveal the driving force of crab metacommunity, Canonical analysis was used to test the relationship between multi‐variables and the pattern of the crab metacommunity. Before the Canonical analysis, we found that the length of the first (main) axis of detrended correspondence analysis (DCA) was 3.92, which represented the community is suited for the redundancy analysis (RDA) based on unweighted linear regression. Prior to conducting an RDA, the presence of a linear trend in the data was determined by testing its significance on the community. Thus, this trend was considered in the RDA. Next, the community matrix is Hellinger‐transformed for selecting environment variables. As for spatial variables, the community matrix is also detrended after been Hellinger‐transformed to avoid the spatial structure being obscured by the linear trend. Then, different variable groups except the spatial linear trend, have, respectively, carried out the forward selection based on the two stopping criteria (Blanchet et al., 2008): (1) the significant level at *α* = .05 and (2) the adjusted *R*‐squared (${R_{\mathrm{adj}}}^{2}$) calculated for all variables in each group. After the RDA, a variance inflation factors (VIF) is used to examine the multicollinearity of all predictors. If a VIF is higher than 10 (in general), the variable should be removed before proceeding with some ecological rules or by variable filtering processes. To ensure the interpretability of the RDA model, an analysis of variance (ANOVA) like permutation test (the number of permutations = 999) was performed for both the joint effect of constraints and each constrained axis.

To explain the importance of each group of variables, the variation partitioning analysis (VPA) and the hierarchical partitioning (HP) were conducted. The VPA uses the ${R_{\mathrm{adj}}}^{2}$ to obtain an unbiased estimate of the proportion of explained variance of each combination of variables (pure environment factors [ENV], pure vegetation factors [VEG], pure detrended spatial factors [GEO], and pure spatial linear trend [TRE] in this research), which is commonly used in the research of biogeography. The *p*‐value of each combination was tested by a series of partial‐RDA models. Then, the HP was performed to test the overall importance of each predictor (Lai et al., 2022), and the *p*‐value for each combination was tested by the permutation test (the number of permutations = 999).

All analysis in this part was conducted using “vegan” (Oksanen et al., 2020) and “rdacca.hp” (Lai et al., 2022) packages.

#### 2.5.4 Distance‐decay relationship and beta diversity partition

The distance‐decay relationship between the beta diversity and the geographic distance reveals both the turnover and nestedness of the structure differentials between communities in space (Morlon et al., 2008; Nekola & White, 1999; Soininen et al., 2007). We divided the beta diversity (*β*_sor_) into turnover (*β*_sim_) and nestedness (*β*_nes_) components based on the formula: *β*_sor_ = *β*_sim_ + *β*_nes_ (Baselga family, Sørensen‐based indices) by using the “beta.div.comp” function of “adespatial” (Dray et al., 2021) packages in R with setting “quant = TRUE” to compute on abundance data. Then the Bray–Curtis similarity equals to 1 − *β*_sor_. Finally, we regressed the similarity, turnover, and nestedness diversities against geographic distance using ordinary least squares models. The relationship was determined by conducting the Spearman correlation test and the significance based on the Mantel statistic. A tringle plot was used to show the relative percentages of the three components between each two plots and represented by the mean value.

#### 2.5.5 Elements of metacommunity structure

The EMS framework is currently one of the best methods for understanding the patterns of metacommunities (Dallas, 2014). A more detailed and comprehensive classification of metacommunity patterns can enhance our understanding of the potential mechanisms underlying metacommunity assembling, beyond those that are based solely on variance partitioning (Heino et al., 2015). The EMS framework is based on three elements: coherence, turnover, and boundary clumping, which are calculated from the presence‐absence interaction matrix (site‐by‐species) that is ordinated via reciprocal averaging. Coherence is measured by counting the embedded absences in the ordinated matrix. Significance is calculated by comparing the observed embedded absences with the expected values of 1000 simulations of the null model through a *z*‐test. Species range turnover is calculated based on the assumption of complete coherence, and the replacement of each species between any two sites is calculated. Significance is also measured through a *z*‐test by comparing it with the null model. Boundary clumping is tested using the chi‐square goodness‐of‐fit test by comparing the observed and expected distribution of range boundaries. If it is significant, then the Morisita's index (*I*) is calculated and compared with 1. The three elements are calculated in order, and depending on the significance of each test, they can be classified into non‐coherent checkerboard and random modes, and coherent nested subsets, evenly spaced, Gleasonian, Clementsian, and their quasi‐modes, see Appendix S1: Section S2 and Figure S1 (Leibold & Mikkelson, 2002; Presley et al., 2010). The quasi‐mode is determined by turnover and only represents a weak degree, which does not affect the typology itself (Presley et al., 2010). The calculation of EMS was completed using the “metacom” package (Dallas, 2014) in R.

#### 2.5.6 Different components of taxonomic diversity and functional diversity among seasons

First, the differences of seasonality in Dongzhaigang were determined by a permutational analysis of multivariate dispersions (PERMDISP) test. Then, we have statistically analyzed four types of functional trait data for the crabs we captured, including (1) body size represented by maximum width, average width, maximum weight, and average weight; (2) diet represented by filter‐feeding, omnivorous, and carnivorous; (3) mobility represented by swimming and crawling types; (4) habitat represented by burrowing and semi‐arboreal types. We calculated the Gower distance of the species‐trait matrix and used “ward.D2” clustering to select the optimal classification cluster based on the silhouette width, and determined the final grouping in combination with ecological significance. The relative abundance and distribution of each functional group were displayed through bar charts and heat maps for each season and the whole year. Based on this, we calculated three functional diversity indices using “FD” (Laliberté et al., 2014; Laliberte & Legendre, 2010) package at the seasonal level: functional dispersion (FDis), functional richness (FGR), and functional redundancy (FRe). FDis is the average distance from each species to the centroid of all species connected by functional traits, considering species abundance (Laliberte & Legendre, 2010). FGR is the functional group richness at the plot level. FRe was calculated following the method suggested by Mouillot et al. (2013):

(5) FRe=SFE

where *S* is the total number of species at a site and FE is the total functional groups at a site. These indices were calculated using the “FD” package. In addition, we also calculated three alpha diversity indices, including Chao1 to characterize species richness, Shannon index, and Simpson index, using the “vegan” package (Oksanen et al., 2020). Analysis of Variance (ANOVA) and Least Significant Difference (LSD) were used for post‐hoc multiple tests to determine the seasonal differences in each index. To explore the influence of aquatic environmental factors on these indices and the relationship between aquatic environmental factors and climatic factors, we calculated Spearman correlations and their significance. To further investigate the contribution of aquatic environmental factors to the changes in each index, we used random forest (based on “randomForest” package; Liaw & Wiener, 2002) to establish regression models and calculated the overall explanatory power and importance of each factor in each model.

## 3 RESULTS

### 3.1 Seasonal differences of aquatic environmental factors

The results of correlation test among aquatic environmental factors showed a high degree of collinearity between total dissolved solids and salinity (Pearson's correlation coefficient = .96, *p* < .001), and only salinity was included in further analysis. PERMDISP test revealed strong evidence (permuted *p*‐value <.001) that all aquatic variables varied significantly among seasons, as shown in Appendix S1: Figure S6.

### 3.2 Additive diversity partitioning of whole year

The results of the survey conducted over four seasons revealed that the overall diversity of Dongzhaigang National Nature Reserve was primarily characterized by significant differences among quadrats, followed by significant differences among the five locations (Table S1). The contribution of diversity within the quadrats was found to be the least (Table S1).

### 3.3 Hierarchical and variation partitioning for redundancy analysis

The environmental, spatial, and vegetation factors were conducted forward selection independently. Finally, the water salinity, the water temperature, and the dissolved oxygen were retained as the environmental factors, the MEM2, MEM3, MEM5, and MEM12 were used as the spatial factors, and three root types (spreading roots, knee roots, and prop roots) were employed as the vegetation factors. The results of the VIF expansion coefficient indicated that all factors range between 1.19 and 6.12, thus, had low redundant constraints and multicollinearity.

**FIGURE 3:**
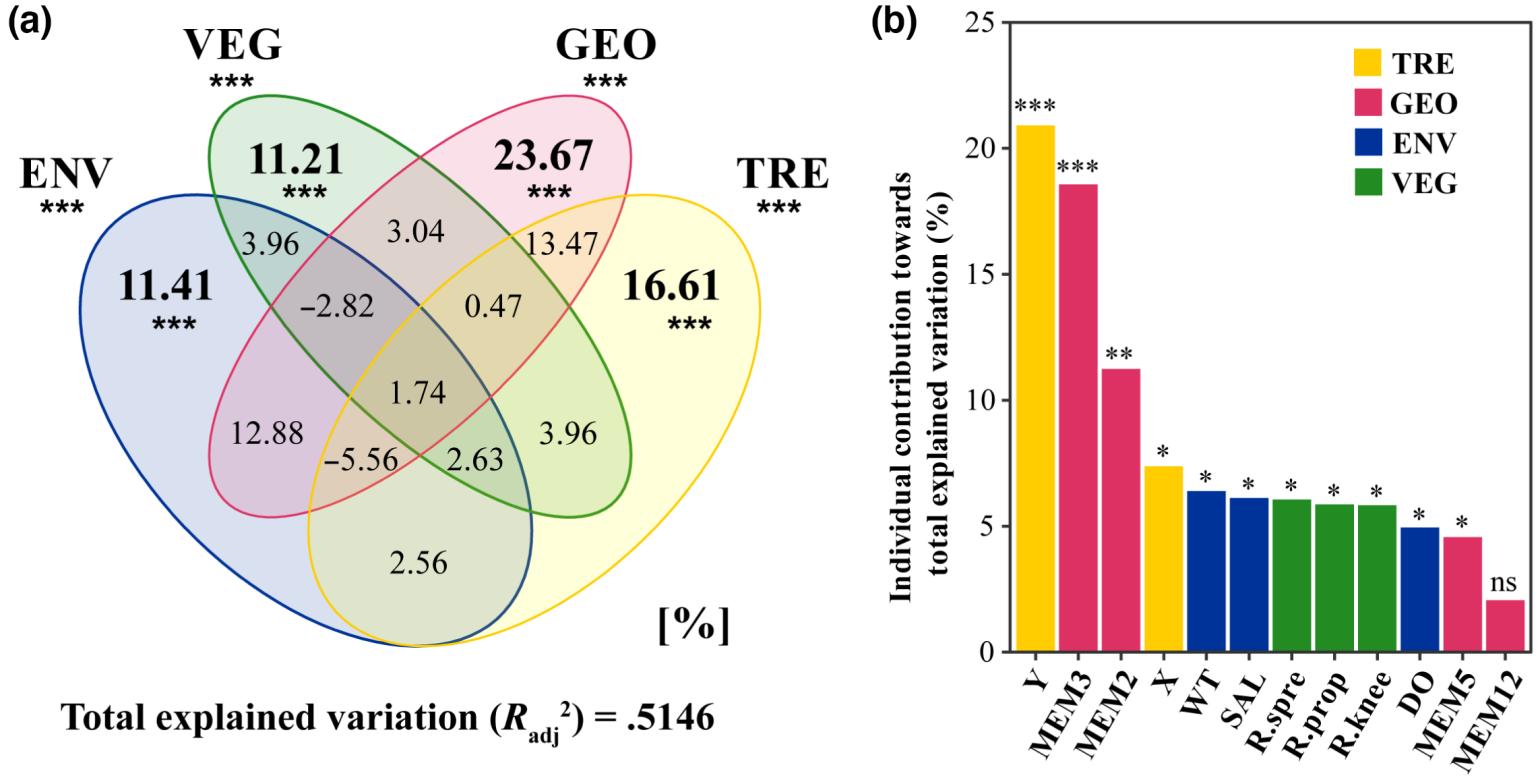
Results of hierarchical and variation partitioning for redundancy analysis of the mangrove crab metacommunity. Variables were selected based on forward selection. (a) Redundancy analysis totally explained 0.5146 variation after adjustment. The Venn plot displays the percentage of total explained variation for each unique part. ENV, environmental factors; GEO, spatial factors based on Moran's Eigenvector Maps (MEM); TRE, linear spatial trends of community structure; VEG, vegetation factors. (b) Hierarchical partitioning shows the individual contribution toward total explained variation. “***”, “**”, “*”, and “ns” indicate very strong evidence (*p* < .001), strong evidence (*p* < .01), moderate evidence (*p* < .05) and litter or no evidence of contribution. DO, water dissolved oxygen; MEM2, 3, 5, 12, Moran's Eigenvector Maps from board scale to fine scale; R.spre, R.prop, R.knee, spreading roots, prop roots, and knee roots; SAL, water salinity; WT, water temperature; X, x‐coordinate; Y, y‐coordinate.

The results of the RDA analysis demonstrated a significant impact of the four factors on the spatial distribution of crab communities (Figure 3a). The ${R_{\mathrm{adj}}}^{2}$ was .5146 and the *p*‐value of the permutation test was <.001. The VPA (Figure 3a) shown that the independent contribution to the explained variance of the spatial factors was 23.67%, while the linear trend was 16.61%. This was higher than the contribution of the water environment (11.41%) and the biological factor (11.21%). The detrended spatial factors (MEMs) and environmental factors accounted for 12.88%, while the vegetation factors and environmental factors accounted for 3.9% (Figure 3a). The results of the analytic hierarchy process identified the explanatory contributions of each factor. The four factors with the highest contributions were spatial linear trends (X/Y coordinate) and broader scale spatial attributes, specifically MEM3 and MEM2. The environmental factors (salinity and water temperature) of the water body were also found to be important, although slightly higher so than the type of vegetation root. In contrast, the fine‐scale attribute MEM12 had the lowest explanatory power with little evidence (Figure 3b).

### 3.4 Distance‐decay relationship and beta‐diversity partitioning

**FIGURE 4:**
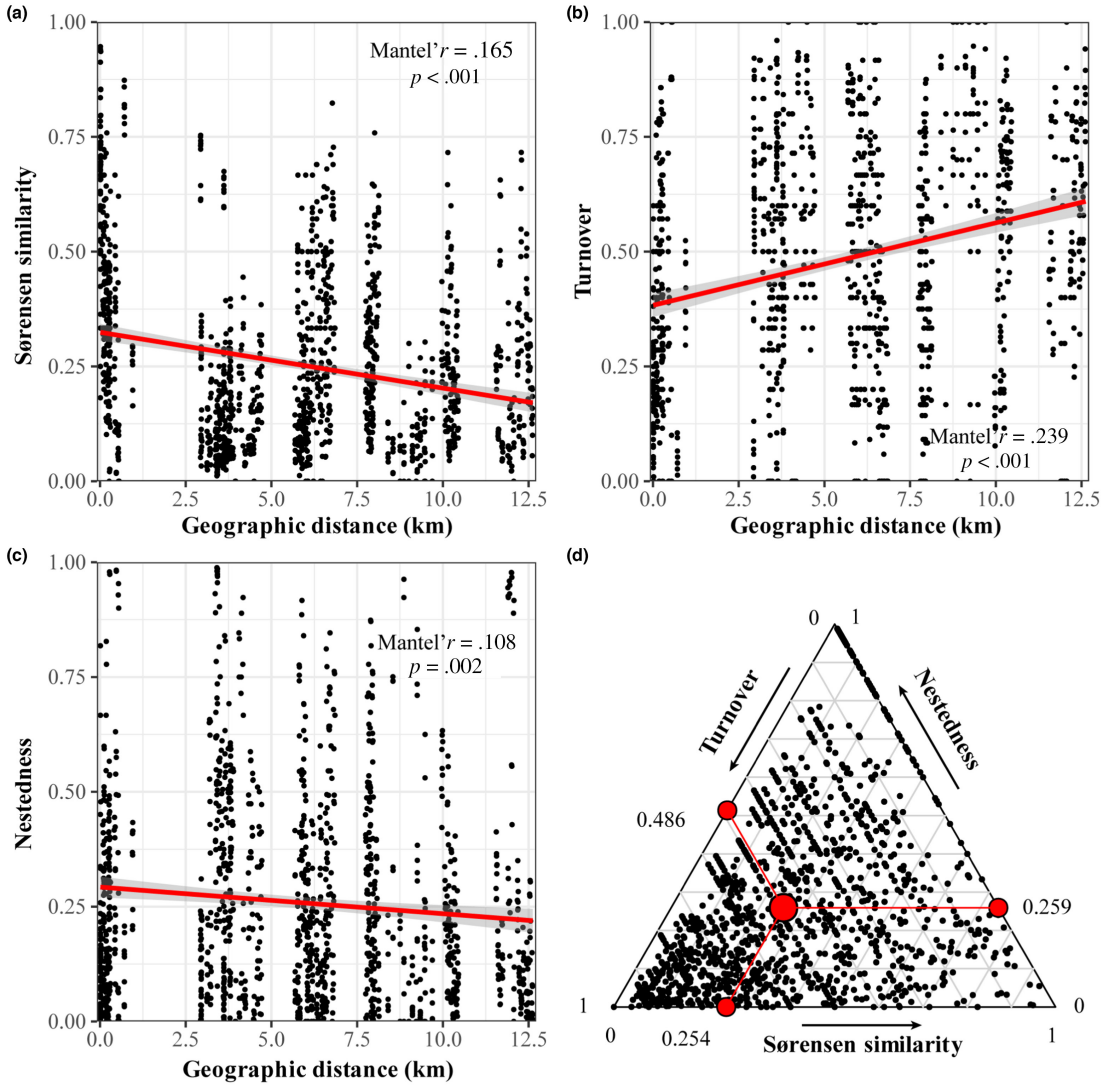
The relationship between geographic distance and (a) Sørensen similarity, (b) turnover component, and (c) nestedness component of beta diversity was analyzed through Spearman correlation test. The significance is based on the Mantel statistic. (d) The triangle plot displays the relative percentages of the three components of beta diversity between each pair of plots, represented by the mean value.

Within the spatial range of 12.6 km, there was very strong evidence that the Sørensen similarity of crab communities declined with the increase of geographical distance (Mantel's *r* = .165, *p* < .001), demonstrating a distance‐decay relationship (Figure 4a). Specifically, the turnover component of beta dissimilarity increased significantly with the increase of distance (Mantel's *r* = .239, *p* < .001) (Figure 4b), accompanied by a significant decline in the nestedness component of the beta dissimilarity with distance (Mantel's *r* = .108, *p* = .002) (Figure 4c). The beta dissimilarity was mainly composed of turnover components (Turnover/Total beta = 65.24%), rather than nestedness components (Nestedness/Total beta = 34.76%). Overall, the spatial difference in crab community diversity was primarily driven by turnover (Figure 4d).

### 3.5 Changes of the elements of metacommunity structure

The analysis of the results in Table 1 reveals the following: (1) both annually and seasonally, significant positive coherence was observed (observed embedded absences lower than simulated mean; *Z* < 0; *p* < .001), indicating the existence of potential environmental gradients in the crab communities within the Dongzhaigang National Nature Reserve. (2) Annually, in summer and spring, significant positive turnover was observed (observed turnover higher than simulated mean; *Z* > 0; *p* < .001), indicating that the structural changes among the communities are higher than expected. However, in autumn and winter, there was no significant positive turnover (observed turnover higher than simulated mean; *Z* > 0; *p* > .05). (3) Finally, analyzing the boundary clumping's Morisita's index (MI), the results indicated that except for the winter season (MI = 1.412, *p* = .162), all other seasons and the overall annual results were significantly >1 (*p* < .05). Thus, results of the summer and spring were consistent with the annual results, showing a Clementsian pattern. The turnover was no longer significant in autumn, but the boundary clumping remained significant, showing a quasi‐Clementsian pattern. In winter, the turnover and boundary clumping were both not significant, but due to the existence of significant positive coherence, it was a quasi‐Gleasonian pattern.

**TABLE 1:** The results of element of metacommunity structure (EMS) analysis of mangrove crab metacommunity across four seasons and the whole year.

Temporal scale	Coherence				Turnover				Clumping		Typology
	Emb, Abs	Sim, mean (SD)	*Z*	*p*	Turnover	Sim, mean (SD)	*Z*	*p*	MI	*p*	
Whole year	579	831 (38)	−6.569	**<.001**	19,281	9905 (2829)	3.314	**<.001**	4.041	**<.001**	Clementsian
Summer	273	581 (36)	−8.343	**<.001**	14,060	8830 (1667)	3.137	**.002**	1.624	**.018**	Clementsian
Autumn	174	334 (26)	−6.069	**<.001**	3972	2888 (807)	1.341	.180	2.179	**.003**	Q‐Clementsian
Winter	64	127 (10)	−6.298	**<.001**	963	783 (258)	0.693	.488	1.412	.162	Q‐Gleasonian
Spring	357	551 (31)	−6.247	**<.001**	7033	3619 (1233)	2.768	**.006**	1.804	**.007**	Clementsian

*Note*: Values in boldface indicate significant differences between observed value and simulated value. Typology reference to the framework described in Presley et al. (2010).

Abbreviations: Emb Abs, observed embedded absences; MI, Morisita's index; Q‐Clementsian, Quasi‐Clementsian pattern; Q‐Gleasonian, Quasi‐Gleasonian patternSim Mean (SD), simulated mean and SD; *Z*, *z*‐score of the *z*‐test.

### 3.6 Seasonal variation of functional structure and alpha diversity

**FIGURE 5:**
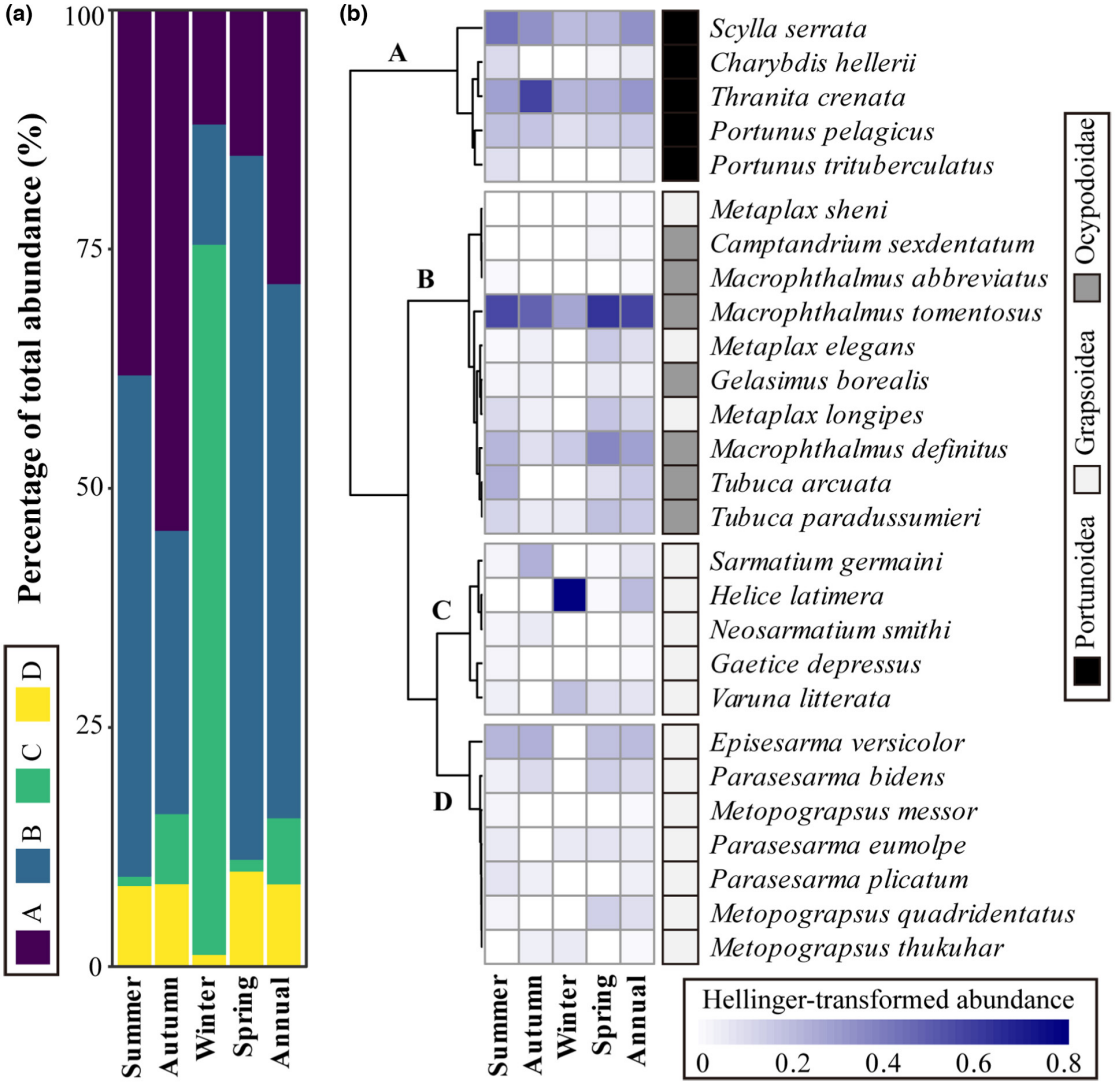
Functional groups of mangrove crabs were defined based on the cluster result of the Gower distance of the species‐traits matrix. (a) Percentage of total abundance of four seasons and the whole year. (b) Heatmap of Hellinger‐transformed abundance of four seasons and the whole year.

The results of clustering based on Gower distance and the silhouette width analysis indicated that crabs can be divided into four functional groups based on their body size, feeding, mobility, and inhabitation (Figure 5, Appendix S1: Figure S7). Based on their ecological roles, crabs were classified into four groups: Group A consisted of larger swimming predators, Group B included crabs belonging to the Ocypodoidae family and the *Metaplax* gene that mainly filter feed on the surface of the substrate, Group C comprised larger crabs belonging to the families Grapsidae, Sesarmidae, and Varunidae, and Group D consisted of arboreal crabs belonging to the family Sesarmidae. The composition and relative proportions of these functional groups varied across different seasons (Figure 5a). The dominant functional groups during spring, summer, and the whole year were C < D < A < B, and the main species contributing to this pattern were *Macrophthalmus tomentosus*, *Scylla serrata*, and *Thranita crenata* (Figure 5). However, in autumn, Group A, especially *Thranita crenata*, was the dominant functional group, while in winter, Group C, mainly due to the contribution of *Helice latimera*, became dominant, and the proportion of other functional groups decreased (Figure 5).

**FIGURE 6:**
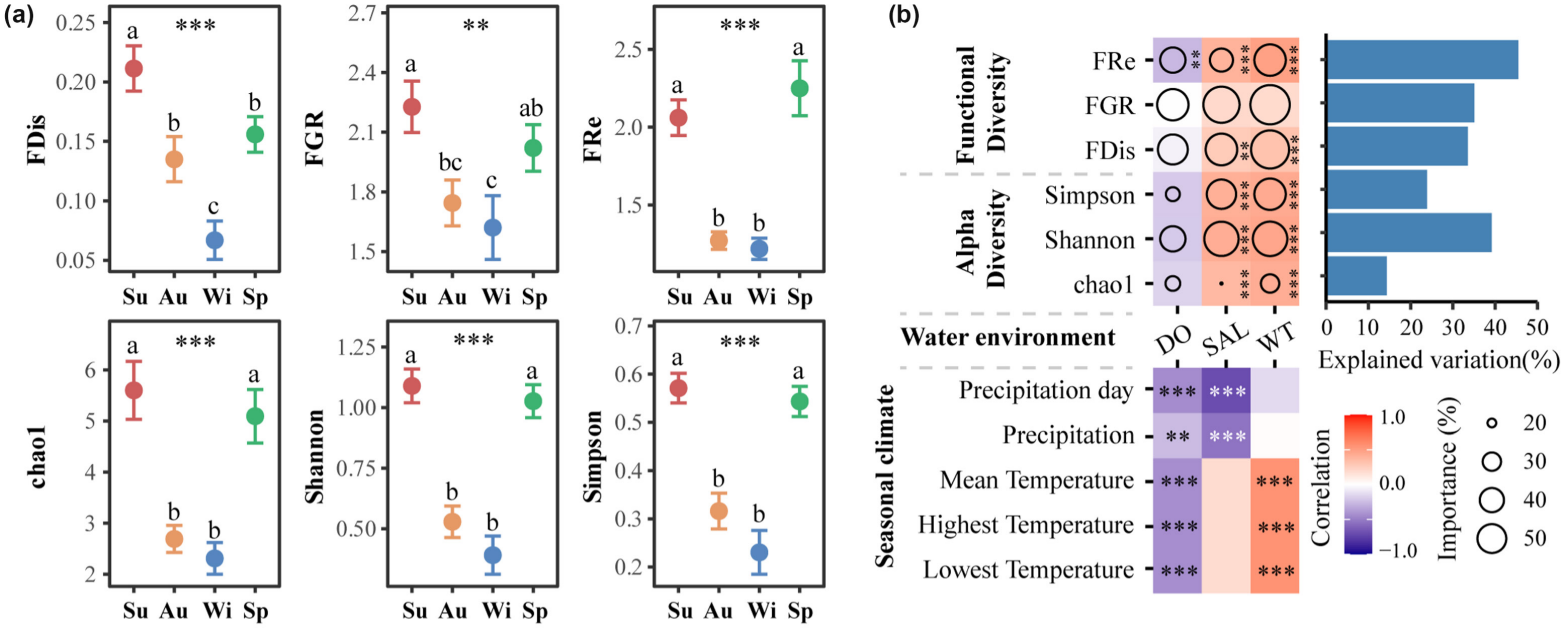
(a) Results of ANOVA and LSD test of functional and taxonomic alpha diversities of mangrove crabs across seasons. The error bars represent the standard error (SE). The same letters indicate no significant difference among the four seasons. (b) Correlation results of Spearman test between water environment factors and diversities or seasonal climate factors. The circle sizes represent the importance of each water environment factor in random forest models. The bar plot displays the total explained variation of random forest models. “***” indicates very strong evidence (*p* < .001). “**” indicates strong evidence (*p* < .01). “*” indicates moderate evidence (*p* < .05). No signal indicates little or no evidence (*p* > .05). Au, autumn; DO, dissolved oxygen; FDis, functional dispersion; FGR, functional richness; FRe, functional redundancy; SAL, water salinity; Sp, spring; Su, summer; Wi, winter; WT, water temperature.

Results of ANOVA and multiple comparison showed significant seasonal differences in functional dispersion, functional group richness, and functional redundancy (Figure 6a). Functional dispersion was significantly higher in summer than in autumn and spring, and significantly lower in winter than in other seasons. Functional group richness did not differ significantly between adjacent seasons but was significantly different between non‐adjacent seasons. Similarly, functional redundancy and alpha diversity of Chao1, Shannon, and Simpson indices showed the same seasonal pattern, with no significant differences between spring and summer or between autumn and winter. But spring and summer were significantly higher than autumn and winter.

Results of Spearman correlation analysis and random forest modeling showed that both functional diversity and taxonomic alpha diversity were significantly associated with seasonal changes in these factors and had a high interpretation rate for functional redundancy and Shannon index (Figure 6b). Dissolved oxygen (DO) had a significant negative correlation with functional redundancy, while salinity and water temperature had significant positive correlations with other community parameters, except for functional groups, and were highly important in the random forest model (Figure 6c). Further analysis of the relationship between the three main water environmental factors and seasonal climate change showed that DO was significantly negatively correlated with precipitation and temperature, while salinity was only significantly negatively correlated with precipitation, and water temperature was only significantly positively correlated with temperature factors.

## 4 DISCUSSION

Global changes, including sea‐level raising and global warming, have resulted in significant modifications to the functional characteristics of crab communities in coastal wetlands (Crotty et al., 2020; Spivak et al., 2019). This shift in potential status, including the transformation into keystone species, has amplified the impact of crabs on coastal wetland vegetation, topography, and the environment, potentially leading to either degradation or prosperity of the wetland vegetation (Crotty et al., 2020). The recent research paradigm highlights the importance of not only herbivorous crabs but also filter‐feeding crabs, thereby increasing the demand for multi‐species, multiscale, and metacommunity research (Cannicci et al., 2008; Xiao et al., 2019, 2021). However, substantial data gaps and a lack of evidence persist in the research on crab metacommunities, hindering a comprehensive understanding of the subject (Lee, 2008; Moore et al., 2020). This study aims to provide new evidence for the study of a bay‐wide scale metacommunity of mangrove crabs in tropical area, and there are several aspects worth exploring.

### 4.1 The Clementsian pattern versus the spatial‐dominated processes

We collected crab community data through surveys in four seasons but analyzed the overall distribution pattern at the annual scale instead of obtaining a comprehensive model through separate analysis in four seasons. This is because the sampling method relies on crab activity, which varies greatly depending on the season, and using an inappropriate overall model could lead to inaccuracies.

Firstly, based on a hierarchical sampling design, the additive decomposition of the gamma diversity of the community indicated a higher contribution of beta diversity, which suggests potential environmental gradients with heterogeneity or spatial restrictions (Appendix S1: Table S1). Therefore, it is necessary to analyze the relative importance of both regional spatial dynamics and local environmental factors.

Second, the results of RDA and HP provide clear evidence. Overall, the water environmental factors and vegetation type factors, which jointly representing local environmental heterogeneity were weaker than the spatial processes that represented by the MEMs and linear trends (Figure 3a). However, their independent explanatory components were all strongly significant. According to the analytical framework of Cottenie (2005), it might be tentatively assumed that the species sorting (SS) and mass effect (ME) patterns coexist. These patterns have been found repeatedly in invertebrates and benthic animals due to their strong reproductive ability and spatial dispersal ability through flight, swimming, or aquatic juvenile stages (Alves et al., 2020; Cottenie, 2005; Heino & Alahuhta, 2015; Li et al., 2021). On the one side, they are subject to local environmental selection and are separated from each other along the environmental gradient, exhibiting the SS paradigm. Since our sampling has a certain hierarchy, adjacent sample plots have stronger communication, and the size of local populations is affected by dispersal, so the ME paradigm also exists. The results of HP showed that the *Y*‐axis in the spatial coordinates was the most important in explaining the metacommunity structure (Figure 3b), which represents the spatial and environmental gradient from the bay mouth to the bay bottom. At the same time, the higher explanatory power of the board‐scale MEMs than the fine scale indicates that long‐distance dispersal limits the similarity between local communities. The short‐distance dispersal of mangrove crabs relies on movement, while the long‐distance dispersal relies on the passive drift of the larvae from the bay mouth to the bay bottom through water flow during rising tide. However, contrary to our conjecture, even within the bay‐wide scale that much more finer (~12.6 km) than the spatial scale in Cottenie (2005) (>1000 km), the dispersal of crab larvae is still limited by terrain and distance. This limitation may be influenced by salinity and temperature of seawater or by predators during the dispersal process (Diele & Simith, 2006; Morgan, 1990; Nurdiani & Zeng, 2007).

We detected two main water environmental factors, salinity, and water temperature, that also significantly affect the spatial pattern of the crab community, similar to other studies (Sharifian et al., 2021; Vahidi et al., 2020). There is evidence suggesting that variations in salinity and water temperature primarily influence the survival, growth rate, and dispersal distance of crab larvae (Anger, 2003; Diele & Simith, 2006; Nurdiani & Zeng, 2007; O'Connor & Epifanio, 1985). At the tidal scale and global scale, changes in salinity also affect the distribution of mangrove crabs (Sharifian et al., 2021; Skov et al., 2002). We also discovered a salinity gradient that influences crab distribution throughout the bay; however, this gradient is not strictly linear, as it is affected by adjacent freshwater sources and effluents from aquaculture ponds. The heterogeneous bottom surface structure created by mangrove roots also significantly impacts crab distribution, yet the distribution and composition of crabs on mudflats appear more random, as rootless types are excluded in the forward selection process. This suggests that mudflats without vegetation may serve as habitats where various species can move or appear, whereas habitats with specialized selection often consist of diverse vegetation structures that offer shelter at the expense of visibility and activity range (Kristensen, 2008). The presence of environmental heterogeneity accounts for the majority of the turnover component in beta diversity decomposition, showing that short‐distance dispersal enhances the similarity of neighboring communities, while the absence of long‐distance dispersal results in species replacement and a decline in similarity (Figures 3 and 4). The results of EMS, reflecting the community pattern based on P/A data, demonstrate that the community exhibits significant coherence (Table 1), characterized by weak competition and the presence of potential environmental gradients. However, we also faced the same conundrum as Meynard et al. (2013), in which, through VPA, we determined that the spatial structure and linear trends of MEMs related to spatial processes point to the dominant role of dispersal limitation. This effect was further corroborated by beta diversity decomposition and distance attenuation. Nonetheless, the EMS results revealed the presence of an overall Clementsian pattern, underscoring the significance of environmental filtering. This finding once again highlights the limitations of the EMS method in uncovering dispersal processes (Presley et al., 2010). Adopting a comprehensive analysis using multiple approaches can effectively address the shortcomings of individual methods (Ovaskainen et al., 2019). This approach also represents the future direction of community analysis, which will become easier to implement due to the widespread application of the R language.

### 4.2 The drivers of the seasonally changes of the crab metacommunity

The results of EMS revealed the seasonal variation in crab metacommunity patterns, closely resembling the differences between dry and rainy seasons in tropical rainforests (Comita et al., 2010). During the study, the crab community experienced fluctuations in autumn and winter, but it regained similar functional diversity, taxonomic alpha diversity, and community patterns in spring as seen in summer (Figure 6a). However, alpha diversity and FRe of autumn and winter were significantly lower than spring and summer, potentially indicating that the community has lower stress resistance, possibly due to seasonal environmental pressures (Figure 6a). The results confirmed the importance of water salinity and water temperature in influencing crab metacommunity patterns, with each factor being separately affected by precipitation and air temperature (Figure 6b). We observed higher amounts of precipitation in autumn and lower air temperatures in winter, leading to changes in the functional composition and ecological niche compression of crabs during these seasons. When extrapolated, this evidence suggests that the impacts of global warming on water temperature and the effects of freshwater or sewage input on water salinity and dissolved oxygen could threaten or alter the structure and function of crab communities (Bartolini et al., 2011; Cannicci et al., 2009; Ellison & Cannicci, 2016). Therefore, cold wave or typhoon‐induced extreme rainfall events are also expected to significantly affecting crab metacommunities, as has already been observed in other benthic fauna such as mangrove mollusks (Chen et al., 2021).

## 5 CONCLUSION

Our study has addressed some knowledge gaps, but future research is still needed to explore crab population patterns across more diverse spatiotemporal scales, as the processes revealed by the metacommunity framework depend on these scales. The new evidence we provided highlights the necessity of considering both spatial processes and local environmental filtering and employing multi‐approach analyses when studying the patterns and underlying mechanisms of crab metacommunities. Crabs are not only economically significant species in their habitat, impacting the livelihoods of local residents, but they also serve as crucial ecosystem engineers. The importance of this group is further heightened under the pressures of global change and urgent ecosystem restoration tasks. Additional research on the metacommunity patterns of crab populations and related driving factors will establish a theoretical and data foundation for predicting future changes in crab communities. Previously, limitations in research methods for crabs have impeded the acquisition of related data. However, with advances in image‐based sampling and artificial intelligence, new methods may change this situation, allowing for more comprehensive data and descriptions of crab communities and promoting both fishery and ecological conservation.

## AUTHOR CONTRIBUTIONS

**Xuan Gu:** Conceptualization (lead); formal analysis (lead); investigation (lead); methodology (lead); visualization (lead); writing – original draft (lead). **Guogui Chen:** Formal analysis (equal); methodology (equal); validation (equal); writing – review and editing (equal). **Yufeng Lin:** Investigation (equal); validation (equal); writing – review and editing (equal). **Wenqing Wang:** Data curation (equal); funding acquisition (equal); validation (equal); writing – review and editing (equal). **Mao Wang:** Data curation (lead); funding acquisition (lead); project administration (lead); resources (lead); supervision (lead); writing – review and editing (lead).

## CONFLICT OF INTEREST STATEMENT

The authors declare that the research was conducted in the absence of any commercial or financial relationships that could be construed as a potential conflict of interest.

## Supporting information


Appendix S1.

## Data Availability

The datasets analyzed for this study (Gu et al., 2023) can be found in the Zenodo repository from doi:10.5281/zenodo.7827643.

## References

[ece310191-bib-0001] Alves, A. T. , Petsch, D. K. , & Barros, F. (2020). Drivers of benthic metacommunity structure along tropical estuaries. Scientific Reports, 10, 1739. 10.1038/s41598-020-58631-1 32015384 PMC6997391

[ece310191-bib-0002] Anger, K. (2003). Salinity as a key parameter in the larval biology of decapod crustaceans. Invertebrate Reproduction and Development, 43(1), 29–45. 10.1080/07924259.2003.9652520

[ece310191-bib-0003] Barbier, E. B. , Hacker, S. D. , Kennedy, C. , Koch, E. W. , Stier, A. C. , & Silliman, B. R. (2011). The value of estuarine and coastal ecosystem services. Ecological Monographs, 81(2), 169–193. 10.1890/10-1510.1

[ece310191-bib-0004] Bartolini, F. , Cimò, F. , Fusi, M. , Dahdouh‐Guebas, F. , Lopes, G. P. , & Cannicci, S. (2011). The effect of sewage discharge on the ecosystem engineering activities of two east African fiddler crab species: Consequences for mangrove ecosystem functioning. Marine Environmental Research, 71(1), 53–61. 10.1016/j.marenvres.2010.10.002 21047678

[ece310191-bib-0005] Baselga, A. (2010). Partitioning the turnover and nestedness components of beta diversity. Global Ecology and Biogeography, 19(1), 134–143. 10.1111/j.1466-8238.2009.00490.x

[ece310191-bib-0006] Bivand, R. S. , & Wong, D. W. S. (2018). Comparing implementations of global and local indicators of spatial association. Test, 27(3), 716–748. 10.1007/s11749-018-0599-x

[ece310191-bib-0007] Blanchet, F. G. , Legendre, P. , & Borcard, D. (2008). Forward selection of explanatory variables. Ecology, 89(9), 2623–2632. 10.1890/07-0986.1 18831183

[ece310191-bib-0008] Cannicci, S. , Bartolini, F. , Dahdouh‐Guebas, F. , Fratini, S. , Litulo, C. , Macia, A. , Mrabu, E. J. , Penha‐Lopes, G. , & Paula, J. (2009). Effects of urban wastewater on crab and mollusc assemblages in equatorial and subtropical mangroves of East Africa. Estuarine, Coastal and Shelf Science, 84(3), 305–317. 10.1016/j.ecss.2009.04.021

[ece310191-bib-0009] Cannicci, S. , Burrows, D. , Fratini, S. , Smith, T. J. , Offenberg, J. , & Dahdouh‐Guebas, F. (2008). Faunal impact on vegetation structure and ecosystem function in mangrove forests: A review. Aquatic Botany, 89(2), 186–200. 10.1016/j.aquabot.2008.01.009

[ece310191-bib-0010] Cannicci, S. , Fusi, M. , Cimó, F. , Dahdouh‐Guebas, F. , & Fratini, S. (2018). Interference competition as a key determinant for spatial distribution of mangrove crabs. BMC Ecology, 18, 8. 10.1186/s12898-018-0164-1 29448932 PMC5815208

[ece310191-bib-0011] Carrasquilla‐Henao, M. , & Juanes, F. (2017). Mangroves enhance local fisheries catches: A global meta‐analysis. Fish and Fisheries, 18(1), 79–93. 10.1111/faf.12168

[ece310191-bib-0012] Chen, G. , Gu, X. , Liu, Y. , Wang, W. , & Wang, M. (2021). Different functional feeding groups of mangrove soil molluscs invoke unique co‐occurrence patterns in response to a climate extreme. Diversity and Distributions, 28(2), 331–345. 10.1111/ddi.13467

[ece310191-bib-0013] Colossi Brustolin, M. , Nagelkerken, I. , Moitinho Ferreira, C. , Urs Goldenberg, S. , Ullah, H. , & Fonseca, G. (2019). Future Ocean climate homogenizes communities across habitats through diversity loss and rise of generalist species. Global Change Biology, 25(10), 3539–3548. 10.1111/gcb.14745 31273894

[ece310191-bib-0014] Comita, L. S. , Muller‐Landau, H. C. , Aguilar, S. , & Hubbell, S. P. (2010). Asymmetric density dependence shapes species abundances in a tropical tree community. Science, 329(5989), 330–332. 10.1126/science.1190772 20576853

[ece310191-bib-0015] Cottenie, K. (2005). Integrating environmental and spatial processes in ecological community dynamics. Ecology Letters, 8(11), 1175–1182. 10.1111/j.1461-0248.2005.00820.x 21352441

[ece310191-bib-0016] Crist, T. O. , Veech, J. A. , Gering, J. C. , & Summerville, K. S. (2003). Partitioning species diversity across landscapes and regions: A hierarchical analysis of α, β, and γ diversity. The American Naturalist, 162(6), 734–743. 10.1086/378901 14737711

[ece310191-bib-0017] Crotty, S. M. , Ortals, C. , Pettengill, T. M. , Shi, L. , Olabarrieta, M. , Joyce, M. A. , Altieri, A. H. , Morrison, E. , Bianchi, T. S. , Craft, C. , Bertness, M. D. , & Angelini, C. (2020). Sea‐level rise and the emergence of a keystone grazer alter the geomorphic evolution and ecology of southeast US salt marshes. Proceedings of the National Academy of Sciences of the United States of America, 117(30), 17891–17902. 10.1073/pnas.1917869117 32661151 PMC7395507

[ece310191-bib-0018] Crowder, L. B. , & Cooper, W. E. (1982). Habitat structural complexity and the interaction between bluegills and their prey. Ecology, 63(6), 1802–1813. 10.2307/1940122

[ece310191-bib-0019] Dallas, T. (2014). Metacom: An R package for the analysis of metacommunity structure. Ecography, 37(4), 402–405. 10.1111/j.1600-0587.2013.00695.x

[ece310191-bib-0020] De Bie, T. , De Meester, L. , Brendonck, L. , Martens, K. , Goddeeris, B. , Ercken, D. , Hampel, H. , Denys, L. , Vanhecke, L. , Van der Gucht, K. , Van Wichelen, J. , Vyverman, W. , & Declerck, S. A. J. (2012). Body size and dispersal mode as key traits determining metacommunity structure of aquatic organisms. Ecology Letters, 15(7), 740–747. 10.1111/j.1461-0248.2012.01794.x 22583795

[ece310191-bib-0021] Diele, K. , & Simith, D. J. B. (2006). Salinity tolerance of northern Brazilian mangrove crab larvae, *Ucides cordatus* (Ocypodidae): Necessity for larval export? Estuarine, Coastal and Shelf Science, 68(3), 600–608. 10.1016/j.ecss.2006.03.012

[ece310191-bib-0022] Diele, K. , Tran Ngoc, D. M. , Geist, S. J. , Meyer, F. W. , Pham, Q. H. , Saint‐Paul, U. , Tran, T. , & Berger, U. (2013). Impact of typhoon disturbance on the diversity of key ecosystem engineers in a monoculture mangrove forest plantation, can Gio biosphere reserve, Vietnam. Global and Planetary Change, 110, 236–248. 10.1016/j.gloplacha.2012.09.003

[ece310191-bib-0023] Diniz, L. P. , Petsch, D. K. , & Bonecker, C. C. (2021). Zooplankton β diversity dynamics and metacommunity structure depend on spatial and temporal scales in a neotropical floodplain. Freshwater Biology, 66(7), 1328–1342. 10.1111/fwb.13719

[ece310191-bib-0024] Dray, S. , Bauman, D. , Blanchet, G. , Borcard, D. , Clappe, S. , Guenard, G. , Jombart, T. , Larocque, G. , Legendre, P. , Madi, N. , & Wagner, H. H. (2021). adespatial: Multivariate Multiscale Spatial Analysis . https://CRAN.R‐project.org/package=adespatial

[ece310191-bib-0025] Dray, S. , Legendre, P. , & Peres‐Neto, P. R. (2006). Spatial modelling: A comprehensive framework for principal coordinate analysis of neighbour matrices (PCNM). Ecological Modelling, 196(3–4), 483–493. 10.1016/j.ecolmodel.2006.02.015

[ece310191-bib-0026] Duke, N. C. , Meynecke, J. O. , Dittmann, S. , Ellison, A. M. , Anger, K. , Berger, U. , Cannicci, S. , Diele, K. , Ewel, K. C. , Field, C. D. , Koedam, N. , Lee, S. Y. , Marchand, C. , Nordhaus, I. , & Dahdouh‐Guebas, F. (2007). A world without mangroves? Science, 317(5834), 41–42. 10.1126/science.317.5834.41b 17615322

[ece310191-bib-0027] Ellison, J. C. , & Cannicci, S. (2016). Impacts and effects of ocean warming on mangrove species and ecosystems. In D. Laffoley & J. M. Baxter (Eds.), Explaining ocean warming: Causes, scale, effects and consequences (1st ed., pp. 135–146). IUCN.

[ece310191-bib-0028] Fu, H. , Zhang, Y. , Ao, X. , Wang, W. , & Wang, M. (2019). High surface elevation gains and prediction of mangrove responses to sea‐level rise based on dynamic surface elevation changes at Dongzhaigang Bay, China. Geomorphology, 334, 194–202. 10.1016/j.geomorph.2019.03.012

[ece310191-bib-0029] Gu, X. , Chen, G. , Lin, Y. , Wang, W. , & Wang, M. (2023). Mangrove crab sampling data in Dongzhaigang National Nature Reserve, Haikou, Hainan Province, China (v1.0) . 10.5281/zenodo.7827644

[ece310191-bib-0030] Heino, J. , & Alahuhta, J. (2015). Elements of regional beetle faunas: Faunal variation and compositional breakpoints along climate, land cover and geographical gradients. Journal of Animal Ecology, 84(2), 427–441. 10.1111/1365-2656.12287 25251566

[ece310191-bib-0031] Heino, J. , Soininen, J. , Alahuhta, J. , Lappalainen, J. , & Virtanen, R. (2015). A comparative analysis of metacommunity types in the freshwater realm. Ecology and Evolution, 5(7), 1525–1537. 10.1002/ece3.1460 25897391 PMC4395181

[ece310191-bib-0032] Heino, J. , & Tolonen, K. T. (2017). Ecological drivers of multiple facets of beta diversity in a lentic macroinvertebrate metacommunity. Limnology and Oceanography, 62(6), 2431–2444. 10.1002/lno.10577

[ece310191-bib-0033] Henry, D. A. W. , & Cumming, G. S. (2016). Spatial and environmental processes show temporal variation in the structuring of waterbird metacommunities. Ecosphere, 7(10), e01451. 10.1002/ecs2.1451

[ece310191-bib-0034] Jabot, F. , Laroche, F. , Massol, F. , Arthaud, F. , Crabot, J. , Dubart, M. , Blanchet, S. , Munoz, F. , David, P. , & Datry, T. (2020). Assessing metacommunity processes through signatures in spatiotemporal turnover of community composition. Ecology Letters, 23(9), 1330–1339. 10.1111/ele.13523 32567194

[ece310191-bib-0035] Kristensen, E. (2008). Mangrove crabs as ecosystem engineers; with emphasis on sediment processes. Journal of Sea Research, 59(1–2), 30–43. 10.1016/j.seares.2007.05.004

[ece310191-bib-0036] Lai, J. , Zou, Y. , Zhang, J. , & Peres‐Neto, P. R. (2022). Generalizing hierarchical and variation partitioning in multiple regression and canonical analyses using the rdacca.Hp R package. Methods in Ecology and Evolution, 13(4), 782–788. 10.1111/2041-210x.13800

[ece310191-bib-0037] Laliberte, E. , & Legendre, P. (2010). A distance‐based framework for measuring functional diversity from multiple traits. Ecology, 91(1), 299–305. 10.1890/08-2244.1 20380219

[ece310191-bib-0038] Laliberté, E. , Legendre, P. , & Shipley, B. (2014). FD: Measuring functional diversity from multiple traits, and other tools for functional ecology . https://cran.r‐project.org/web/packages/FD/index.html 10.1890/08-2244.1 20380219

[ece310191-bib-0039] Lamy, T. , Wisnoski, N. I. , Andrade, R. , Castorani, M. C. N. , Compagnoni, A. , Lany, N. , Marazzi, L. , Record, S. , Swan, C. M. , Tonkin, J. D. , Voelker, N. , Wang, S. , Zarnetske, P. L. , & Sokol, E. R. (2021). The dual nature of metacommunity variability. Oikos, 130(12), 2078–2092. 10.1111/oik.08517

[ece310191-bib-0040] Lande, R. (1996). Statistics and partitioning of species diversity, and similarity among multiple communities. Oikos, 76(1), 5–13. 10.2307/3545743

[ece310191-bib-0041] Lee, S. Y. (1998). Ecological role of grapsid crabs in mangrove ecosystems: A review. Marine and Freshwater Research, 49(4), 335–343. 10.1071/MF97179

[ece310191-bib-0042] Lee, S. Y. (2008). Mangrove macrobenthos: Assemblages, services, and linkages. Journal of Sea Research, 59(1), 16–29. 10.1016/j.seares.2007.05.002

[ece310191-bib-0043] Lee, S. Y. , Primavera, J. H. , Dahdouh‐Guebas, F. , McKee, K. , Bosire, J. O. , Cannicci, S. , Diele, K. , Fromard, F. , Koedam, N. , Marchand, C. , Mendelssohn, I. , Mukherjee, N. , & Record, S. (2014). Ecological role and services of tropical mangrove ecosystems: A reassessment. Global Ecology and Biogeography, 23(7), 726–743. 10.1111/geb.12155

[ece310191-bib-0044] Leibold, M. A. , Chase, J. M. , & Ernest, S. K. M. (2017). Community assembly and the functioning of ecosystems: How metacommunity processes alter ecosystems attributes. Ecology, 98(4), 909–919. 10.1002/ecy.1697 27984663

[ece310191-bib-0045] Leibold, M. A. , Holyoak, M. , Mouquet, N. , Amarasekare, P. , Chase, J. M. , Hoopes, M. F. , Holt, R. D. , Shurin, J. B. , Law, R. , Tilman, D. , Loreau, M. , & Gonzalez, A. (2004). The metacommunity concept: A framework for multi‐scale community ecology. Ecology Letters, 7(7), 601–613. 10.1111/j.1461-0248.2004.00608.x

[ece310191-bib-0046] Leibold, M. A. , & Mikkelson, G. M. (2002). Coherence, species turnover, and boundary clumping: Elements of meta‐community structure. Oikos, 97(2), 237–250. 10.1034/j.1600-0706.2002.970210.x

[ece310191-bib-0047] Leung, J. Y. S. (2015). Habitat heterogeneity affects ecological functions of macrobenthic communities in a mangrove: Implication for the impact of restoration and afforestation. Global Ecology and Conservation, 4, 423–433. 10.1016/j.gecco.2015.08.005

[ece310191-bib-0048] Li, Z. , Heino, J. , Song, Z. , Jiang, X. , Wang, J. , Liu, Z. , Chen, X. , Meng, X. , Zhang, J. , & Xie, Z. (2021). Spatio‐temporal variation of macroinvertebrate metacommunity organization in a monsoon‐climate region. Journal of Biogeography, 48(12), 3118–3130. 10.1111/jbi.14270

[ece310191-bib-0049] Liaw, A. , & Wiener, M. (2002). Classification and regression by randomForest. R News, 2(3), 18–22.

[ece310191-bib-0050] Liu, S. , Cui, Z. , Zhao, Y. , & Chen, N. (2022). Composition and spatial‐temporal dynamics of phytoplankton community shaped by environmental selection and interactions in the Jiaozhou Bay. Water Research, 218, 118488. 10.1016/j.watres.2022.118488 35489150

[ece310191-bib-0051] Logue, J. B. , Mouquet, N. , Peter, H. , & Hillebrand, H. (2011). Empirical approaches to metacommunities: A review and comparison with theory. Trends in Ecology and Evolution, 26(9), 482–491. 10.1016/j.tree.2011.04.009 21641673

[ece310191-bib-0052] Marrec, R. , Pontbriand‐Pare, O. , Legault, S. , & James, P. M. A. (2018). Spatiotemporal variation in drivers of parasitoid metacommunity structure in continuous forest landscapes. Ecosphere, 9(1), e02075. 10.1002/ecs2.2075

[ece310191-bib-0053] McLain, D. K. , & Pratt, A. E. (2010). Food availability in beach and marsh habitats and the size of the fiddler crab claw, a sexually selected weapon and signal. Oikos, 119(3), 508–513. 10.1111/j.1600-1706.2009.18105.x

[ece310191-bib-0054] Meynard, C. N. , Lavergne, S. , Boulangeat, I. , Garraud, L. , Van Es, J. , Mouquet, N. , & Thuiller, W. (2013). Disentangling the drivers of metacommunity structure across spatial scales. Journal of Biogeography, 40(8), 1560–1571. 10.1111/jbi.12116 24790288 PMC4000944

[ece310191-bib-0055] Moore, A. , Fauset, E. , & Asher, F. (2020). Consumer impacts on ecosystem functions in coastal wetlands: The data gap. Ecosphere, 11(2), e03042. 10.1002/ecs2.3042

[ece310191-bib-0056] Morgan, S. G. (1990). Impact of Planktivorous fishes on dispersal, hatching, and morphology of estuarine crab larvae. Ecology, 71(5), 1639–1652. 10.2307/1937574

[ece310191-bib-0057] Morlon, H. , Chuyong, G. , Condit, R. , Hubbell, S. , Kenfack, D. , Thomas, D. , Valencia, R. , & Green, J. L. (2008). A general framework for the distance‐decay of similarity in ecological communities. Ecology Letters, 11(9), 904–917. 10.1111/j.1461-0248.2008.01202.x 18494792 PMC2613237

[ece310191-bib-0058] Mouillot, D. , Graham, N. A. J. , Villéger, S. , Mason, N. W. H. , & Bellwood, D. R. (2013). A functional approach reveals community responses to disturbances. Trends in Ecology and Evolution, 28(3), 167–177. 10.1016/j.tree.2012.10.004 23141923

[ece310191-bib-0059] Nekola, J. C. , & White, P. S. (1999). The distance decay of similarity in biogeography and ecology. Journal of Biogeography, 26(4), 867–878. 10.1046/j.1365-2699.1999.00305.x

[ece310191-bib-0060] Nobbs, M. , & Blamires, S. J. (2017). Fiddler crab spatial distributions are influenced by physiological stressors independent of sympatric interactions. Journal of Experimental Marine Biology and Ecology, 491, 19–26. 10.1016/j.jembe.2017.03.007

[ece310191-bib-0061] Nurdiani, R. , & Zeng, C. (2007). Effects of temperature and salinity on the survival and development of mud crab, *Scylla serrata* (Forsskål), larvae. Aquaculture Research, 38(14), 1529–1538. 10.1111/j.1365-2109.2007.01810.x

[ece310191-bib-0062] O'Connor, N. J. , & Epifanio, C. E. (1985). The effect of salinity on the dispersal and recruitment of fiddler crab larvae. Journal of Crustacean Biology, 5(1), 137–145. 10.2307/1548226

[ece310191-bib-0063] Oksanen, J. , Blanchet, F. G. , Friendly, M. , Kindt, R. , Legendre, P. , McGlinn, D. , Minchin, P. R. , O'Hara, R. B. , Simpson, G. L. , Solymos, P. , Stevens, M. H. H. , Szoecs, E. , & Wagner, H. (2020). vegan: Community Ecology Package . R package version 2.5–7. https://CRAN.R‐project.org/package=vegan

[ece310191-bib-0064] Ovaskainen, O. , Rybicki, J. , & Abrego, N. (2019). What can observational data reveal about metacommunity processes? Ecography, 42(11), 1877–1886. 10.1111/ecog.04444

[ece310191-bib-0065] Peres‐Neto, P. R. , & Legendre, P. (2010). Estimating and controlling for spatial structure in the study of ecological communities. Global Ecology and Biogeography, 19(2), 174–184. 10.1111/j.1466-8238.2009.00506.x

[ece310191-bib-0066] Peres‐Neto, P. R. , Legendre, P. , Dray, S. , & Borcard, D. (2006). Variation partitioning of species data matrices: Estimation and comparison of fractions. Ecology, 87(10), 2614–2625. 10.1890/0012-9658(2006)87[2614:Vposdm]2.0.Co;2 17089669

[ece310191-bib-0067] Presley, S. J. , Higgins, C. L. , & Willig, M. R. (2010). A comprehensive framework for the evaluation of metacommunity structure. Oikos, 119(6), 908–917. 10.1111/j.1600-0706.2010.18544.x

[ece310191-bib-0068] R Core Team . (2021). R: A language and environment for statistical computing. R Foundation for Statistical Computing.

[ece310191-bib-0069] Sharifian, S. , Kamrani, E. , & Saeedi, H. (2021). Global future distributions of mangrove crabs in response to climate change. Wetlands, 41, 1–14. 10.1007/s13157-021-01503-9

[ece310191-bib-0070] Shih, H. T. , Ng, P. K. L. , Fang, S. H. , Chan, B. K. K. , & Wong, K. J. H. (2010). Diversity and distribution of fiddler crabs (Brachyura: Ocypodidae: *Uca*) from China, with new records from Hainan Island in the South China Sea. Zootaxa, 2640(1), 1–19. 10.11646/zootaxa.2640.1.1

[ece310191-bib-0071] Siqueira, T. , Bini, L. M. , Roque, F. O. , Marques Couceiro, S. R. , Trivinho‐Strixino, S. , & Cottenie, K. (2012). Common and rare species respond to similar niche processes in macroinvertebrate metacommunities. Ecography, 35(2), 183–192. 10.1111/j.1600-0587.2011.06875.x

[ece310191-bib-0072] Skov, M. W. , Vannini, M. , Shunula, J. P. , Hartnoll, R. G. , & Cannicci, S. (2002). Quantifying the density of mangrove crabs: Ocypodidae and Grapsidae. Marine Biology, 141, 725–732. 10.1007/s00227-002-0867-9

[ece310191-bib-0073] Soininen, J. , Heino, J. , & Wang, J. (2018). A meta‐analysis of nestedness and turnover components of beta diversity across organisms and ecosystems. Global Ecology and Biogeography, 27(1), 96–109. 10.1111/geb.12660

[ece310191-bib-0074] Soininen, J. , McDonald, R. , & Hillebrand, H. (2007). The distance decay of similarity in ecological communities. Ecography, 30(1), 3–12. 10.1111/j.2006.0906-7590.04817.x

[ece310191-bib-0075] Spivak, A. C. , Sanderman, J. , Bowen, J. L. , Canuel, E. A. , & Hopkinson, C. S. (2019). Global‐change controls on soil‐carbon accumulation and loss in coastal vegetated ecosystems. Nature Geoscience, 12, 685–692. 10.1038/s41561-019-0435-2

[ece310191-bib-0076] Thompson, P. L. , Guzman, L. M. , De Meester, L. , Horváth, Z. , Ptacnik, R. , Vanschoenwinkel, B. , Viana, D. S. , & Chase, J. M. (2020). A process‐based metacommunity framework linking local and regional scale community ecology. Ecology Letters, 23(9), 1314–1329. 10.1111/ele.13568 32672410 PMC7496463

[ece310191-bib-0077] Vahidi, F. , Fatemi, S. M. R. , Danehkar, A. , Mashinchian, A. , & Musavi Nadushan, R. (2020). Benthic macrofaunal dispersion within different mangrove habitats in Hara biosphere reserve, Persian gulf. International Journal of Environmental Science and Technology, 17, 1295–1306. 10.1007/s13762-019-02469-2

[ece310191-bib-0078] Wang, M. , Gao, X. Q. , & Wang, W. Q. (2014). Differences in burrow morphology of crabs between Spartina alterniflora marsh and mangrove habitats. Ecological Engineering, 69, 213–219. 10.1016/j.ecoleng.2014.03.096

[ece310191-bib-0079] Wang, W. , Fu, H. , Lee, S. Y. , Fan, H. , & Wang, M. (2020). Can strict protection stop the decline of mangrove ecosystems in China? From rapid destruction to rampant degradation. Forests, 11(1), 55. 10.3390/f11010055

[ece310191-bib-0080] Wu, W. , Lu, H. P. , Sastri, A. , Yeh, Y. C. , Gong, G. C. , Chou, W. C. , & Hsieh, C. H. (2018). Contrasting the relative importance of species sorting and dispersal limitation in shaping marine bacterial versus protist communities. ISME Journal, 12, 485–494. 10.1038/ismej.2017.183 29125596 PMC5776463

[ece310191-bib-0081] Xiang, H. , Li, K. , Cao, L. , Zhang, Z. , & Yang, H. (2020). Impacts of pollution, sex, and tide on the time allocations to behaviours of *Uca arcuata* in mangroves. Science of the Total Environment, 742, 140609. 10.1016/j.scitotenv.2020.140609 32721739

[ece310191-bib-0082] Xiao, K. , Wilson, A. M. , Li, H. , & Ryan, C. (2019). Crab burrows as preferential flow conduits for groundwater flow and transport in salt marshes: A modeling study. Advances in Water Resources, 132, 103408. 10.1016/j.advwatres.2019.103408

[ece310191-bib-0083] Xiao, K. , Wilson, A. M. , Li, H. L. , Santos, I. R. , Tamborski, J. , Smith, E. , Lang, S. Q. , Zheng, C. M. , Luo, X. , Lu, M. Q. , & Correa, R. E. (2021). Large CO_2_ release and tidal flushing in salt marsh crab burrows reduce the potential for blue carbon sequestration. Limnology and Oceanography, 66(1), 14–29. 10.1002/lno.11582

