# Supplementary material for: Drivers of the spatiotemporal patterns of the mangrove crab metacommunity in a tropical bay
Source: Ecol Evol. 2023 Jun 14;13(6):e10191. doi: 10.1002/ece3.10191 (PMC10266579; doi:10.1002/ece3.10191)
Supplement: Supplementary file 1 — Appendix S1. [file ECE3-13-e10191-s001.docx]

**Appendix S1 for:**

Drivers of the spatio-temporal patterns of the mangrove crab metacommunity in a tropical bay

**Xuan Gu^1, 2, 3^, Guogui Chen^1, 4, 5^, Yufeng Lin^1, 2, 3^, Wenqing Wang^1, 2, 3^, Mao Wang^1, 2, 3*^**

^1^Key Laboratory of the Ministry of Education for Coastal and Wetland Ecosystems, College of the Environment & Ecology, Xiamen University, Xiamen, China

^2^Zhangjiang Estuary Mangrove Wetland Ecosystem Station, National Observation and Research Station for the Taiwan Strait Marine Ecosystem, Xiamen University, Zhangzhou, China

^3^Engineering Research Center of Fujian Province for Coastal Wetland Protection and Ecological Recovery, College of the Environment & Ecology, Xiamen University, Xiamen, China

^4^State Key Laboratory of Water Environmental Simulation, School of Environment, Beijing Normal University, Beijing, China

^5^Research and Development Center for Watershed Environmental Eco-Engineering, Advanced Institute of Natural Sciences, Beijing Normal University, Zhuhai, China

*** Corresponding author:**Mao Wang
[wangmao@xmu.edu.cn](mailto:wangmao@xmu.edu.cn)

Section S1: Four paradigms of metacommunities (Leibold et al., 2004)

**Patch dynamics**: This paradigm focuses on the importance of local environmental conditions, and how species interactions within individual patches shape community dynamics. It emphasizes the idea that coexistence is possible given a competition-colonization trade-off.

**Species sorting**: This paradigm emphasizes the role of environmental filtering and local abiotic features in shaping community composition, where species that are adapted to similar environmental conditions are more likely to coexist. It also highlights the importance of dispersal limitation in restricting the movement of species between habitats.

**Mass effect**: This paradigm focuses on the role of dispersal in shaping community dynamics, particularly the idea that the movement of individuals and populations between habitats can create "mass effects" that alter community composition and dynamics. This paradigm also highlights the importance of regional species pools in shaping local community assembly.

**Neutral**: This paradigm assumes that all species in a community are functionally equivalent and that their interactions are governed by random processes such as birth, death, and dispersal. It highlights the idea that neutral processes can explain observed patterns of community composition and dynamics, without the need to invoke species-specific interactions or environmental filtering. One influential paper in this paradigm is Hubbell (2001), "The unified neutral theory of biodiversity and biogeography."

Leibold, M. A., Holyoak, M., Mouquet, N., Amarasekare, P., Chase, J. M., Hoopes, M. F., Holt, R. D., Shurin, J. B., Law, R., Tilman, D., Loreau, M., and Gonzalez, A. (2004). The metacommunity concept: A framework for multi-scale community ecology. *Ecology Letters* 7(7)**,** 601-613. https://doi.org/10.1111/j.1461-0248.2004.00608.x.

Section S2: Elements of metacommunity structure


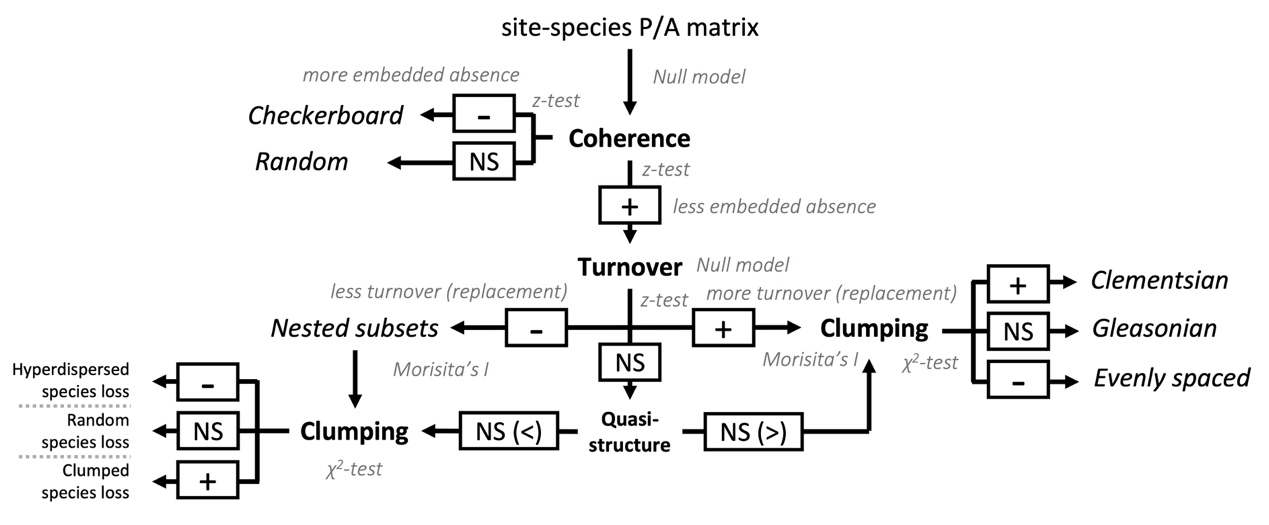


Figure S1 The comprehensive framework of a pattern-based approach to describe the idealized metacommunity structure, proposed by Presley et al. (2010) (in black) and we add some details (in grey).

Elements of metacommunity structure is a pattern-based framework for analyzing community structure based on three elements of species distribution: (a) coherence, (b) turnover, and (c) boundary clumping. These elements can be determined by the double ordination of the species incidence matrix (coherence and turnover) and Morisita’s index (boundary clumping). The significance of each element is tested by z-test or ﻿χ^2^ goodness-of-fit test.

These elements can form six different types of metacommunity structure:

- Random structure: Species distribution shows no pattern, and there is no evident environmental gradient or spatial structure.
- Checkerboard structure: Species distribution shows a pattern of mutual exclusion or negative correlation, possibly due to strong competition or other biotic interactions.
- Nested structure: Species distribution shows a hierarchical pattern, where sites with higher species richness contain all the species of sites with lower richness, possibly due to environmental filtering or incomplete colonization. Presley et al. (2010) distinguished three forms of nestedness: nested subsets, nestedness resulting from species loss or gain.
- Evenly spaced structure: Species distribution shows coherence and boundary clumping, but no turnover, possibly due to spatial isolation or limited dispersal ability.
- Gleasonian gradient structure: Species distribution shows coherence and turnover, but no boundary clumping, possibly due to individualistic responses to a primary environmental gradient.
- Clementsian gradient structure: Species distribution shows coherence, turnover and boundary clumping, possibly due to the existence of several distinct community types that have similar responses along an environmental gradient.

Presley et al. (2010) defined quasi-patterns indicates a weaker degree but the same pattern of nested, evenly spaced, Gleasonian and Clementsian structure. These structures can reflect the mechanisms underlying community structure and dynamics in different ecosystems.

Leibold, M. A., and Mikkelson, G. M. (2002). Coherence, species turnover, and boundary clumping: Elements of meta-community structure. *Oikos* 97(2)**,** 237-250. https://doi.org/10.1034/j.1600-0706.2002.970210.x.

Presley, S. J., Higgins, C. L., and Willig, M. R. (2010). A comprehensive framework for the evaluation of metacommunity structure. *Oikos* 119(6)**,** 908-917. https://doi.org/10.1111/j.1600-0706.2010.18544.x.


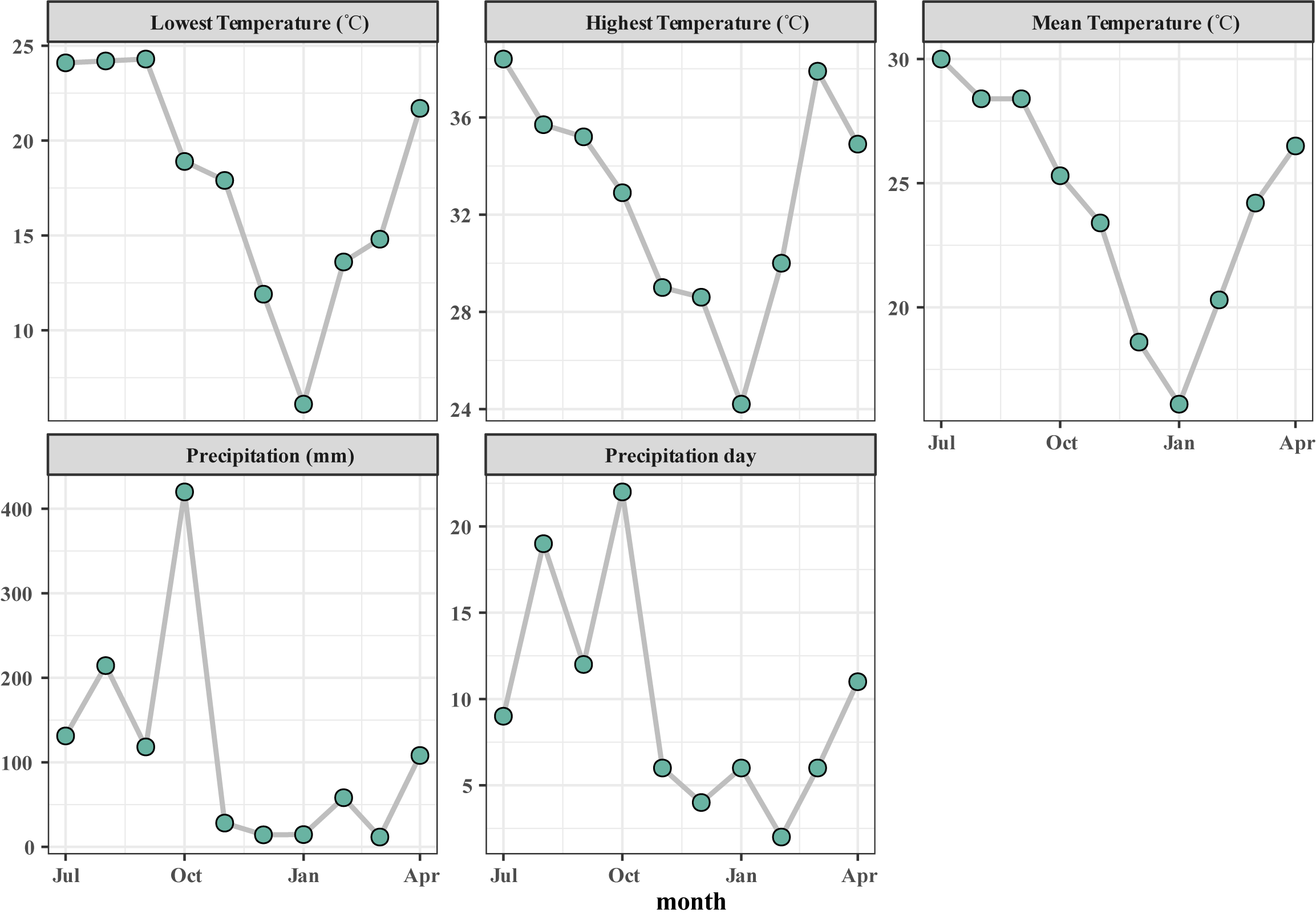


Figure S2 The air temperature and precipitation trends in the Dongzhaigang National Nature Reserve from July 2020 to April 2021.


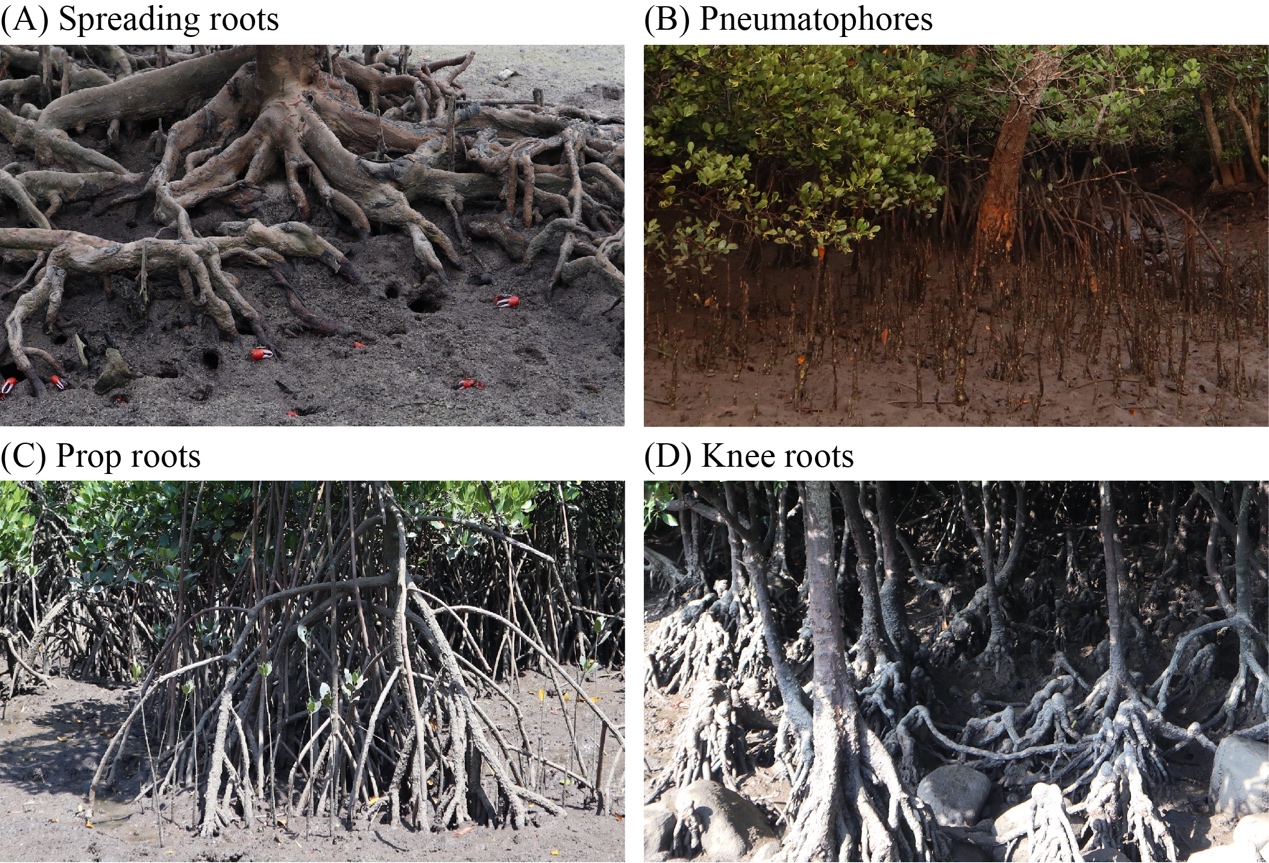


Figure S3 Different root types of mangroves.


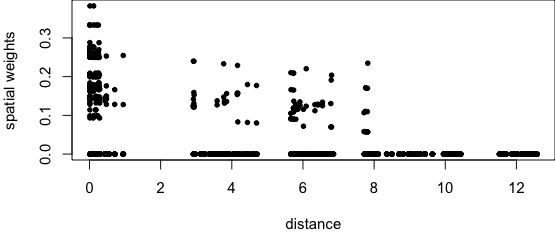


Figure S4 Scatter plot of the relationship between spatial weighting and distance when α=2 in concave-down function (*f*_2_).


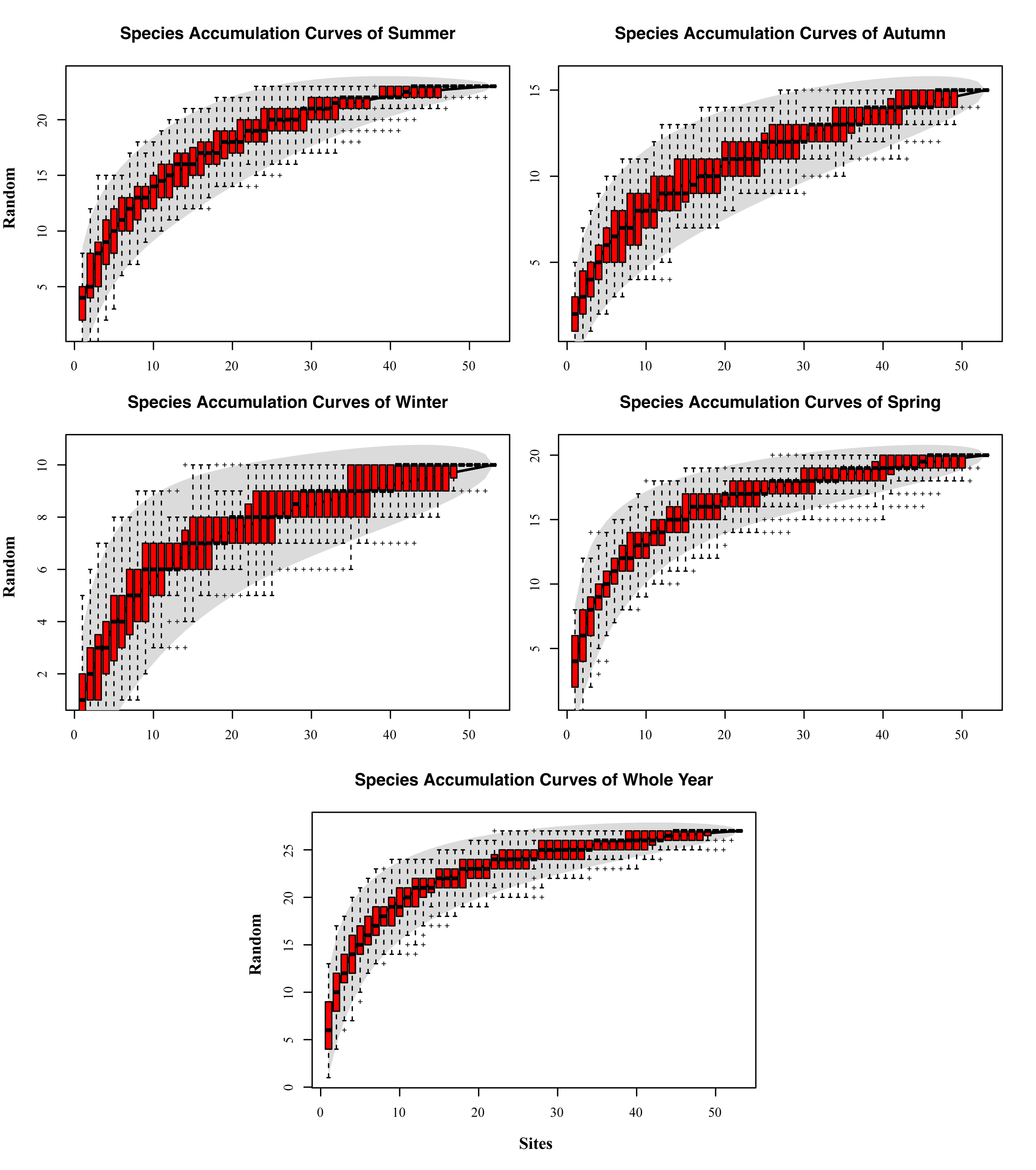


Figure S5 Species accumulation curves of four seasons and whole year describe the completeness of sampling.


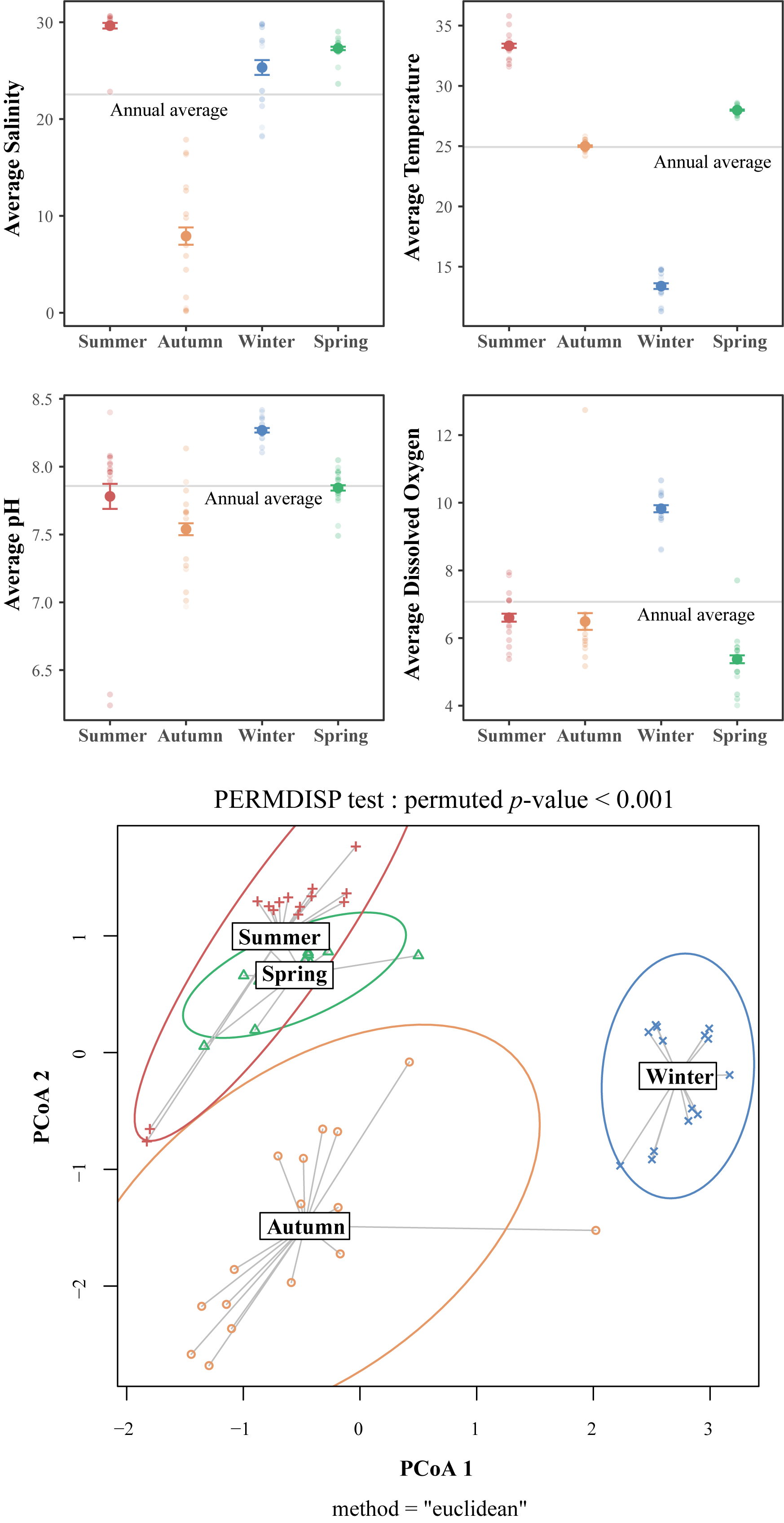


Figure S6 The results of the seasonal trends in four aquatic environmental factors and the permutational analysis of multivariate dispersions (PERMDISP) test showed significant differences across the four seasons. The error bars represent the standard error (SE).


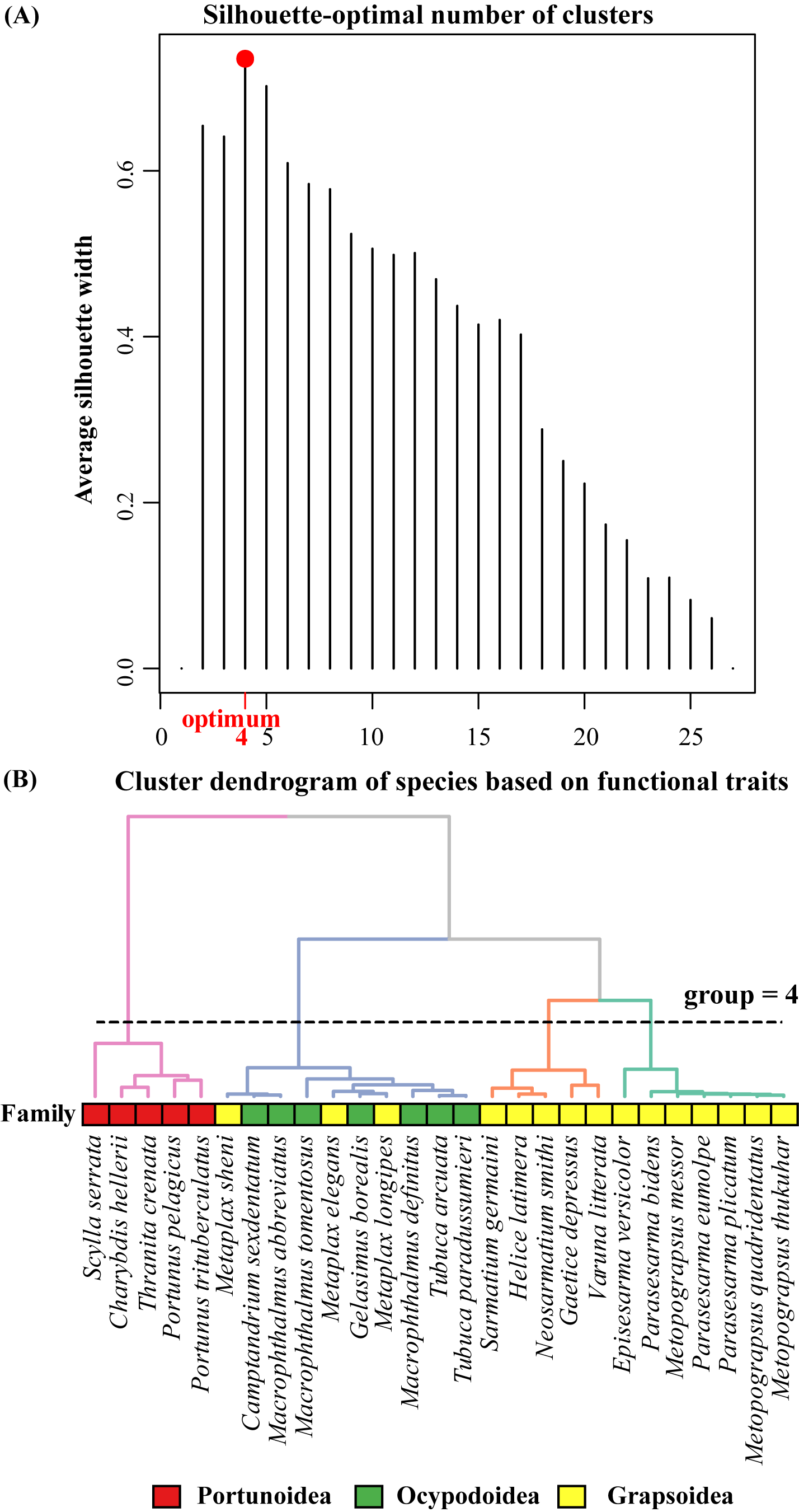


Figure S7 Cluster analysis of crab traits based on Gower distance. (A) The silhouette width indicates that four categories are the optimal classification. (B) Crabs classified into four functional groups and their respective families.

Table S1 The results of additive diversity partitioning

| Diversity | Observed | SES | Simulated | *P* value |
| --- | --- | --- | --- | --- |
| gamma | 27 |  |  |  |
| alpha | 8.32 | -27.01 | 13.86 | <0.001*** |
| beta among quadrats | 10.21 | 3.54 | 8.66 | <0.001*** |
| beta among sites | 8.47 | 9.81 | 4.48 | <0.001*** |
